# A model for the human fetal ventricular myocyte electrophysiology

**DOI:** 10.1371/journal.pcbi.1013889

**Published:** 2026-01-27

**Authors:** Adelisa Avezzú, Stefano Longobardi, Anita Alvarez-Laviada, Francisca Schultz, Julia Gorelik, Catherine Williamson, Steven A. Niederer

**Affiliations:** 1 Department of Biomedical Engineering, School of Biomedical Engineering and Imaging Sciences, King’s College London, London, United Kingdom; 2 National Heart and Lung Institute, Imperial College London, London, United Kingdom; 3 Department of Metabolism, Digestion and Reproduction, Imperial College London, London, United Kingdom; Nanjing University, CHINA

## Abstract

Fetal cardiac arrhythmias can lead to stillbirth, but direct studies on the human fetal heart are challenging. To address this, we developed a computational model of human fetal ventricular myocyte (hfVM) electrophysiology, focusing on early gestation (10 weeks). This model incorporates major ionic currents, including fetal-specific T-type calcium and funny currents, and is calibrated using mRNA expression data and experimental measurements. The hfVM model replicates key electrophysiological features, such as a shorter action potential duration and a more positive resting membrane potential compared to adult cells. Global sensitivity analysis reveals that the resting membrane potential is primarily influenced by the funny current and I_K1_, while action potential repolarisation depends mainly on I_Kr_. Additionally, the sarcoplasmic reticulum contributes to calcium release, but less so than in adults; instead, the T-type calcium current and the sodium-calcium exchanger are more prominent in initiating calcium transients. This is the first human fetal ventricular myocyte model available for studying fetal cardiac physiology, pathology, and potential pharmacological interventions. It provides novel insights into the dominant ion channels governing fetal electrophysiology and calcium dynamics, offering a foundation for understanding arrhythmias and guiding therapeutic strategies.

## Author summary

Irregular heart rhythms in unborn babies can sometimes lead to stillbirth. However, studying these conditions directly in human fetuses is challenging. To address this, we developed a computer-based model that simulates how fetal heart cells function during early pregnancy. This model helps us understand how electrical signals and calcium levels interact to regulate the heartbeat in developing babies. By identifying which cellular components are most influential, we can better comprehend how certain disruptions might lead to dangerous heart rhythms. Our findings offer valuable insights into fetal heart development and could inform future treatments aimed at preventing arrhythmias during pregnancy. This research represents a step forward in safeguarding fetal health and reducing the risk of stillbirth.

## Introduction

Fatal arrhythmias affect 1–2% pregnancies. While the majority have a benign course, in a small proportion of cases they can cause sudden, unpredictable, and otherwise unexplained fetal deaths [1]. Stillbirth, here defined as pregnancy loss from 20 weeks of gestation, complicates 2–6 per 1000 total births in high-income countries [2–9] and occurs with an even higher frequency (~22/1000) for certain ethnic subgroups, such as African Americans [10]. The risk of stillbirth peaks at 20–23 gestational weeks (gw), and increases again after 37 gw, reaching its maximum towards term [10,11]. More than 10% of stillbirths remain unexplained, a proportion that increases up to 24% when the fetal loss occurs after 38 gw [12]. Unexplained still births may be due to fetal arrhythmias. There is evidence that these can be caused by ion channelopathies [1,8,13–17] or specific maternal disorders, including intrahepatic cholestasis of pregnancy (ICP) [18] and pregestational diabetes [19–21]. In all cases the effects of changes in channel kinetic or conduction need to be interpreted in the context of the whole system of ion channels contributing to cardiac myocyte electrophysiology.

In adult cardiac myocyte electrophysiology physics and physiology constrained mathematical models provide a framework for integrating and interpreting channel properties to simulate emergent cellular physiology [22–25]. For human fetal myocyte electrophysiology, this is particularly powerful as we can combine the inherently sparse experimental data.

The aim of this study was to develop the first mathematical model of the healthy human fetal ventricular myocyte (hfVM) designed to represent Ca^2+^ dynamics and cell electrophysiology. The model is derived from the extensively used Ten Tusscher and Panfilov model of the human adult ventricular epicardial myocyte (TT2) [22]. This model provides an important step forward in the investigation of fetal arrhythmia and other fetal diseases related to pregnancy and in the understanding of human fetal heart electrophysiology, of which so far little is known. It can be used as a base and a comparison for the development of future models of human fetal and neonatal myocytes.

## Methods

### Ethics statement

Ventricular cardiomyocytes were isolated from 7 hearts obtained from human fetuses aged 12–17 weeks of gestation terminated (STOP, surgical termination of pregnancy) due to genetic abnormalities (Table 1) after prior written informed consent of the mother using Biobank ethical approval (Imperial College Healthcare Tissue and Biobank licence 12275, REC approval 12/WA/0196) [26]. For the experimental procedure for cell isolation see S2 Text.

**Table 1:** Characteristics of the fetal hearts collected for experiments of Ca^2+^ transient and T-type Ca^2+^ recording and of measurement of cell capacitance.

Heart	Week of Gestation [w + d]	Abnormality	Notes
heart 1	13	trisonomy 13	
heart 2	12	trisonomy 18	
heart 3	16 + 6	trisonomy 21	*
heart 4	14 + 4	trisonomy 13	scan abnormalities
heart 5	14 + 4	trisonomy 21	
heart 6	16 + 5	chromosomal deletion 12q1-2	hydrops
heart 7	12 + 5	Scan abnormalities in lower abdomen	

### Model development

The hfVM model aims to represent healthy human fetal ventricular myocyte Ca^2+^ dynamics and electrophysiology at 10 gw. The hfVM model is based on the Ten Tusscher and Panfilov model for the human adult ventricular myocyte (TT2) [22]. We selected the TT2 model because it is one of the most extensively used ventricular models and for its simplicity relative to other human ventricular myocyte models, as we do not have access to sufficient data to constrain more complex human ventricular myocyte modelling frameworks (such as Grandi [23], O’Hara [24] and Tomek [25]). All the membrane transporters in the adult cell model (both on the cell surface and on the sarcoplasmic reticulum) are assumed to be present in the fetal myocyte model. We assume that channel, pump and transporter dynamics remain unchanged between the adult and fetal myocytes and that differences in electrophysiology and Ca^2+^ dynamics are explained by changes in proteins density, quantitatively represented in the model by the channel conductance or maximum pump or transporter flux. The same assumption was made for Ca^2+^ buffers, i.e., we considered the same buffering proteins as in TT2 but with different density. In addition to the ion channels present in the TT2 adult myocyte model we also introduced the T-type Ca^2+^ channel and the funny current, which are reported in fetal myocytes but are not expressed in adult myocytes. We used the formulations for T-type current initially from both the Demir [27] and the Dokos [28] models for the rabbit sinoatrial node. We used the formulation from the Loewe-Lutz-Fabbri-Severi model [29] of the human sinoatrial node to simulate the funny current, which is implemented as the sum of Na^+^ and K^+^ components. We assumed the same ratio between Na^+^ and K^+^ conductance as in Loewe-Lutz-Fabbri-Severi. The three intracellular compartments described in TT2, sub-sarcolemmal space (SS), cytoplasmic volume (V_c_) and SR are also considered in the hfVM model but with a different distribution of volumes. The total volume, membrane capacitance and basic cycle length (heart rate) in the fetal myocyte model are adapted to fetal values. The equations for the model are described in S1 Text. The T-type Ca^2+^ conductance was obtained by fitting the T-type Ca^2+^ channel to voltage-clamp ventricular myocytes T-type current recordings from human fetal hearts at 12–17 gw. The remaining parameters are constrained by mRNA measurements and fitted to human fetal AP data of 8–11 gw [30] and to experimental Ca^2+^ transient optical recordings from 12-17 gw human fetuses. Due to the inherent challenges in working with human fetal heart tissue there is a scarcity of available data. For this reason we calibrated the model to a range of data sources: direct experimental measurements of T-type Ca^2+^ channels and Ca^2+^ concentration in human fetal ventricular myocytes, data bases of fetal mRNA measurements in human fetal and adult hearts, literature measurements of human fetal heart rates [31], cell geometry [32], and ventircular AP [30].

### Direct experimental measurements

The membrane capacitance was measured in 10 ventricular myocytes coming from hearts 1–3 (Table 2) giving a mean ± S.E.M. value of c_m_ = 13.22 ± 3.33 pF for the average gestational time of 13.5 weeks.

**Table 2:** Cells analysed for T-type Ca^2+^ transient experiments and their measured capacitance.

Heart	Cell	Membrane capacitance [*pF*]
heart 1	cell 1	13.5
	cell 2	12
	cell 3	15
	cell 4	10.5
	cell 5	16
heart 2	cell 6	9
	cell 7	10
	cell 8	10.2
heart 3	cell 9	20
	cell 10	16

#### T-type calcium current.

Ten ventricular cardiomyocytes from three of the seven fetal hearts (Table 2) were used to perform T-type Ca^2+^ current recording. The cells were incubated in a 6 mM Ca^2+^ solution containing nifedipine to suppress the L-type Ca^2+^ current with minimal effects on T-type current. Currents were elicited in voltage-clamp protocol from a holding potential of -90 mV to test potentials ranging from -50 mV to +50 mV in 5 mV increments over 150 ms, at room temperature (20–22 °C degrees). In some cases the nifedipine did not succeed in fully blocking the L-type current. Those cells which exhibited an I-V curve with maximum peak at positive voltages, characteristic of L-type current and in contrast to the T-type current I-V peak at negative voltages [33], were excluded from further analysis (for further details see S2 Text). Analysis was performed on the remaining 5 cells.

#### Internal calcium concentration.

From the 7 hearts described above, fetal ventricular cardiomyocytes and fibroblasts were isolated and separated. Cardiomyocytes were then prepared as described in S2 Text and cultured for up to 10 days. The cells were washed with an external solution containing 141.7 mM Na, 1.3 mM Ca, 5.8 mM K and electrically stimulated. The majority of the cells activated at a frequency of 1.5 Hz which were selected for analysis, in order to be able to adapt the model to a specific pacing period to fit it to the experimental Ca^2+^ transient curves. The Ca^2+^ transient was recorded using optical fluorescence, in arbitrary units. The measured Ca^2+^ transients from the 7 cardiomyocytes were averaged and the mean internal Ca^2+^ curve used to fit the simulated Ca^2+^ transient at different depolarisation and repolarisation times, specifically, t50_up_ (half depolarisation time), t_peak_, t25, t50, t75, t90 (where the number indicates the percentage of repolarisation). Internal resting Ca^2+^ was set to be less than 200 nM and the Ca^2+^ transient amplitude was set to be less than 1 µM [34,35].

### Experimental data from the literature

Additional characteristic values of the fetal heart at 10 gw, such as heart rate and cell volume, were acquired from the literature, either directly or by estimate using data at different gestational weeks.

#### Heart rate.

The fetal heart contracts rhythmically as a consequence of the activity of peacemaker cells as early as 3 weeks post-conception. The human fetal heart rate is faster than the adult: it is 110 beats per minute (bpm) at 5–6 weeks of gestation and increases progressively to around 170 bpm at 9–10 weeks followed by a decrease to 150 bpm by 14 weeks, 140 bpm by 20 weeks and 130 bpm at term on average [31,36,37], with variability range from 110 to 180 bpm at 20 gw and 110–160 at term [38]. The reference heart rate of the fetal heart at 10 gw was set at 170 bpm [31], which corresponds to a cycle length of 353 ms and a frequency of 2.8 Hz.

#### Fetal ventricular myocyte volume.

During the embryonic and fetal stages, the number of myocytes increases considerably [32,39–41], then mitotic events gradually decrease and almost stop before birth [40,42]. This mitotic activity does not cease completely through the whole neonatal to adult period [43,44] when heart maturation is caused almost exclusively by an increase in myocyte volume [41,42]. There is an increase in myocyte volume growth rate towards term [32,44,45], when the ventricular myocyte volume is about 1500–2000 µm^3^ [32]. The cardiac myocyte shape changes from spherical in very early gestation to that of an elongated cylinder in neonatal and adult heart [42,46] resulting in a decrease in surface-to-volume ratio, with cylindrical proportions [44,47] that resemble adult cells after 6 months of life [47,48].

Measurements of fetal ventricular myocytes volume were taken from previously reported experimental recordings [32]. Data from a study that included 36 hearts from fetuses in the last two trimesters of pregnancy (12–36 gw) were used. All the fetuses exhibited normal growth and the hearts were considered normal. The volume of the cardiac myocytes was obtained using the dissector method [49] avoiding the over counting of binucleated cells. Volume measurements corresponding at different gestational time points were interpolated using a first order polynomial in order to obtain ventricular myocyte growth rate in the last two trimesters. This relationship was used to estimate the myocyte volume at 10 gw.

#### Cell action potential.

We fit the fetal electrophysiology model using data from one study validated it against two further studies.

##### Calibration data:

In the first study (Study 1) [30], the hfVM model simulated AP was fitted to APs recorded from ventricular myocytes of human fetal hearts at 8–11 weeks of gestation to control measurements in a study with two experimental groups. In both groups the hearts were obtained after surgical termination of pregnancy from healthy people with no negative health factors in the history of their pregnancy. The experiments were carried out in an external solution with 147.5 mM Na^+^, 2 mM Ca^2+^ and 4 mM K^+^ at a temperature of 36 °C and the myocardium samples were stimulated electrically at a frequency of 1 Hz for 1 ms and the stimulation was carried on for 30 minutes before the measurements were taken. The study consisted of two groups. The first group (Group A) consisted of 61 cardiomyocytes from 13 preparations from 7 hearts. The second study (Group B) consisted of 33 ventricular myocytes from 8 preparation obtained from 4 fetal hearts. The action potential duration (APD) at different voltage levels, 0 V, -20 V, -40 V, -60 V, and maximum voltage and resting membrane potential were measured and reported as mean ± S.E.M. (Table 3). The mean values from Group A and B and mean S.T.D were then obtaided with the formula described in [50] and S.E.M. = S.T.D./ √N, with N total numer of samples from Groups A + B.

**Table 3:** Resting membrane potential (RMP), maximum voltage (Vmax) and action potential duration (APD) at given voltage levels (0, -20, -40, -60 mV) of AP recorded from human fetal ventricular myocytes at 8-11 gw from the two different experimental groups analysed in study 1 [30] and their weighed average.

	Group A		Group B		Average	
	n = 61		n = 33		n = 98	
	mean	± SEM	mean	± SEM	mean	± SEM
**RMP** [mV]	-76.4	1.4	-76.7	0.7	-76.5053	1.5652
**V**_**max**_ [mV]	31.1	2.1	30.2	1.7	30.7840	2.7019
**APD**_**V0**_ [ms]	104.5	6.7	108.4	7.5	105.8691	10.0568
**APD**_**V-20**_ [ms]	120.4	5.6	127.7	7.6	122.9628	9.4403
**APD**_**V-40**_ [ms]	131.4	6.3	142.0	8.1	135.1213	10.2616
**APD**_**V-60**_ [ms]	145.5	6.4	159.4	9.3	150.3798	11.2894

##### Validation data:

In the second study (Study 2) [51], APs were measured in spontaneously beating human fetal ventricular myocytes prepared using 37 hearts of 7–12 week old human embryos, obtained from healthy women after surgical interruption of pregnancy. The fetal hearts were extracted and perfused with tyrode solution containing 150 mM Na^+^, 4 mM Ca^2+^ and 5 mM K^+^ at 25 °C. Isolated hearts were activated and spontaneously contracted at a rate of 50–132 bpm (mean 91 bpm). AII records of ventricular transmembrane potential were performed with the whole heart and obtained from superficial fibres on the epicardium of both ventricles. AP amplitude varied from 95 to 120 mV (mean 110 mV) with maximum voltage between 18–30 mV (means 25 mV) and APD in the range of 185–310 ms.

In the third study (Study 3) [52],fetal hearts were obtained from 12 fetuses of 7–12 gw, taken via surgical termination of pregnancy from healthy women with no negative factors in their pregnancy history. Ventricular myocardium samples were maintained and the experiments were carried in a physiological solution with 147.9 mM Na^+^, 2 mM Ca^2+^ and 4 mM K^+^ at 36 °C. APs were recorded at different stimulation frequencies corresponding to cycle lengths of 200, 300, 500, 1000, 2000 and 3000 ms and the APD at different voltages (0 mV, -20 mV, -40 mV, -60 mV) and at 95% repolarisation as wells as resting membrane potential (RMP) and maximum voltage were measured (Table 4).

**Table 4:** Resting membrane potential (RMP), maximum voltage (Vmax) and action potential duration (APD) at given voltage levels (0, -20, -40, -60 mV) and at 95% repolarisation of APs recorded from human fetal ventricular myocytes at 7-12 gw at the stimulation frequency of 1 Hz reported in study 3 [52].

	mean	± SEM
	n = 446	
**RMP** [mV]	-79.37	0.34
**V**_**max**_ [mV]	32.71	0.57
	**n = 35**	
**APD**_**V0**_ [ms]	120.0	5.7
**APD**_**V-20**_ [ms]	144.0	6.0
**APD**_**V-40**_ [ms]	160.0	6.7
**APD**_**V-60**_ [ms]	190.0	9.2
**APD95** [ms]	258.0	17.7

### mRNA expression data

To further constrain the space where to search the parameters to fit the model, we collected data of mRNA expression from 3 online databases. Database 1 [53] reported measurements of genes expression in 2 different fetal ventricles as well as 2 fetal atria at 13 gw and in 2 adults from a mix of ventricular and atrial tissue. Database 2 [54] reported gene expression data in one fetal and two adults from a mix of ventricle and atrial tissue and in one adult ventricle, but with no reference to the gestational age of the fetus. Finally Database 3 [55] contained mRNA expression data from fetal ventricles at 9 and 16 gw. We combined the information obtained from these databases with fetal mRNA data reported in the literature [56,57]

### Model calibration: Cell anatomy

#### Cell volume.

To estimate the fetal cell volume at 10 gw (near the end of the first trimester), we used ventricular myocytes volume growth rate measured in the 2^nd^ and 3^rd^ trimesters [32] and imposed a linear regression to obtain an estimated total cell volume of 935.6 µm^3^ at 10 gw. This compares with the total cell volume in TT2 of 46250 µm^3^ (computed as V_tot_ = c_m_/ (C_m_ x S_v_), where c_m_ is the cell capacitance, C_m_ the cell capacitance per unit surface area and S_v_ the surface to volume ratio as in TT2).

#### Cell membrane capacitance.

We did not have direct measurements of total cell capacitance at 10 gw. Total cell capacitance (c_m_) is equal to C_m_ x S_v_ x V_tot,_ where C_m_ is the membrane capacitance per unit area of the gross cell geometry or C_m_ = c_m_/A_geo_, S_v_ is the surface to volume, assuming the cell geometry to be a cylinder, and V_tot_ is the total volume of the cell, computed as described above. We then take the specific membrane capacitance (C_sc_) which is defined as C_sc_ = c_m_/A_cap_, where A_cap_ is the actual cell surface area, accounting for membrane folding in T-tubules and grooves. We can then write A_cap_/A_geo_ = C_m_/C_sc_, where C_sc_ is assumed to be 1 µF/cm^2^ [58–60], giving a numerical value of C_m_ = A_cap_/A_geo_.

In fetal myocytes T-Tubules are absent in the early immature cell [61,62]. During pregnancy the fetal cell membrane forms invaginations which turn into t-tubules coupled with the SR at around 32 gestational weeks, after which they appear to be similar to the adult. In the TT2 model C_m_ = 2 µF/cm^2^, i.e. A_cap_/A_geo_ = 2, meaning that in the adult myocyte the capacitive area is considered double the geometrical surface area. As T-Tubules are already well developed at 32 gw we assumed A_cap_/A_geo_ matures to its adult value of 2 at 36 gw and starts at a value of 1 at 0 gw. Imposing a linear increase, we obtained C_m_ = 1.278 µF/cm^2^ at 10 gw. For the surface to volume ratio (S_v_), we assumed the fetal cell to reach same cylindrical proportions as in adult (same as in TT2) at 6 months after birth (see introduction [47,48]), while we computed the S_v_ ratio at 13.5 gw from the experimentally measured cell capacitance. Imposing a linear regression between these two time points we estimated S_v_ at 10 gw. The cell capacitance in the hfVM model was then obtained as c_m_ = C_m_ x S_v_ x V_tot_ and is 11.8234 pF, which compares with a capacitance of 185 pF in the TT2 model.

#### Intracellular volumes.

In TT2 the total intracellular volume considered for ionic movement is constituted of V_c_ (cytoplasmic volume), V_SS_ (sub-sarcolemmal space) and V_SR_ (sarcoplasmic reticulum). In total these three volumes make up 38% of the whole cell volume. We maintained the same proportion in our fetal model but with different relative contributions of the three intracellular spaces to account for underdevelopment of cell membrane and SR at an early fetal stage. Both sarcoplasmic reticulum and cell membrane T-tubules seem to resemble adult myocytes at 30–32 gw [61]. We assumed fetal myocyte volumes are fully developed at 36 gw. This is consistent with evidence that the SR is fully functioning in the neonatal ventricular myocyte. We then assumed that V_SS_ and V_SR_ develop linearly in time and rescaled them of a factor α _volume_ linear in time, starting with no T-tubules or SR (α _volume_ = 0) at week 0 and reaching mature proportions (α _volume_ = 1) of 0.12% V_tot_, and 2.37% V_tot_, respectively, taken from the TT2 cell, at 36 gw. This allowed us to calculate the volume of the cytosol as the remaining volume, needed to reach 38% of the total cell volume at mature proportions. In this way we have V_C_ = 348.677 µm^3^, V_SR_ = 6.164 µm^3^ and V_SS_ = 0.312 µm^3^ at 10 gw (obtained for α _volume_ = 0.278).

### Model calibration: ion channels, membrane pumps and transporters, calcium buffers density

During human fetal heart development, the expression and function of various ion channels and transporters undergo significant changes. The fast Na^+^ current (I_Na_) increases as Na^+^ channel expression rises, leading to a higher maximum dV/dt and greater action potential (AP) amplitude [63]. Similarly, the inward rectifier K^+^ current (I_K1_) becomes more prominent, contributing to a more negative resting membrane potential as the fetus matures [64]. The rapid delayed rectifier K^+^ current (I_Kr_), mediated by hERG channels, maintains stable mRNA levels from early gestation through adulthood [65]. The slow delayed rectifier K^+^ current (I_Ks_) also exhibits consistent expression throughout development [65]. L-type Ca^2+^ current (I_CaL_) expression is low at 8 weeks of gestation but increases until birth, continuing to rise into adulthood [56,66]. Conversely, T-type Ca^2+^ current (I_CaT_) is predominant in fetal ventricular myocytes and diminishes until after birth [63,67], suggesting a crucial role in early cardiac contraction when sarcoplasmic reticulum function is underdeveloped. The sodium-calcium exchanger (I_NaCa_) shows increasing protein expression during gestation, peaking near birth, and declining postnatally [57,68]. The sodium-potassium pump current (I_NaK_) activity varies among species, with some showing increased activity postnatally [69], while others exhibit a decrease in specific subunit expressions [70–72]. The transient outward K^+^ current (I_to_) increases after birth in several species [68]. In humans, infants have about half the Ito current density compared to adults, indicating a limited role in fetal heart electrophysiology [73]. The funny current (I_f_) is present in fetal and neonatal ventricular myocytes but disappears in adult ventricles, remaining only in pacemaker cells [74,75].

After imposing the fetal cell parameters that could be measured, inferred or estimated directly, we calibrated the remaining ion channel, pumps, transporter and Ca^2+^ buffer densities to approximate the 10 gw fetal ventricle myocyte. In each case we simulated the experimental protocols, matching temperature in the Nernst potential, pacing rate and extracellular ionic concentrations. As we had access to detailed experimental measurements of T-type current recordings in voltage-clamp, we fitted the T-type Ca^2+^ channel density directly. To fit the model to experimental data we also imposed threshold values for internal ionic concentrations enforcing internal Na to stay within a physiological limit of 16 mM [76,77].

#### T-type current fit.

Previously Demir [27] and Dokos [28] published T-type current models for the rabbit sinoatrial node. These two structures have been widely used and use Hodgkin-Huxley equations. While the L-type channel was pharmacologically blocked in our measurements, there was evidence of a residual L-type current. To account for this, we considered both T-type only and both T-type and L-type current models when interpreting the measurements. We considered Demir, Dokos and TT2 L-type formulations. The recorded current-time curves were divided by the respective membrane capacitance then fitted with 8 model combinations: 2 with only T-type current and 6 with both T-type and L-type. When a step voltage change is applied, it takes a few hundred microseconds for this to be sensed by the cell. We introduced the onset time as a free variable in the fitting to account for this delay. The conductance of each channel was varied over a fixed range. For each conductance value the onset time t_0_ was fitted. For any single voltage the normalized L^2^ error *E* was computed and the total error *E*_*tot*_ for a single cell was obtained as the weighted average error on all voltage steps *V*_*j*_*, j = 1,..,v*, where v is the number of applied voltage steps.


$$\begin{array}{c} E_{tot}=\;\sum {\mathrm{\backslash nolimits}}_{j}\frac{\text{1}}{v}w_{j}E\left(V_{j}\right),\;\;E\left(V_{j}\right)=\frac{\sqrt{\sum_{i}{\left(I_{exp}\left(t_{i}\right)-I_{sim}\left(t_{i}\right)\right)}^{\text{2}}}}{\sqrt{\sum_{i}{\left(I_{exp}\left(t_{i}\right)\right)}^{\text{2}}}},\;\;w_{j}=\frac{I_{peak}\left(V_{j}\right)}{\underset{i}{\text{max}}I_{peak}\left(V_{i}\right)} \end{array}$$

The model combination and corresponding conductance that produce the smallest error over all the other model combinations and conductance values was chosen as the best fit. We then used the change in expression of T-type Ca^2+^ channel genes in mRNA Database 3 (fetal data at 9 and 16 gw), which is consistent T-type current variation over the same range reported in literature [56], is used to extrapolate the conductance value at 10 gw from the one obtained with the T-type current fit which is representative of fetal data at 13 gw.

#### Calcium transient and action potential fit.

Having fixed the T-type channel conductance in the model the remaining parameters to be fitted are the following membrane channels, transporters and pumps conductance: g_Na_, g_CaL_, g_to_, g_Kr_, g_Ks_, g_K1_, k_NaCa_, p_NaK_, g_pCa_, g_pK_, g_bNa_, g_bCa_, g_fK_, Ca^2+^ buffering parameters for troponin C and calsequestrin (a_TropC_, a_CASQ_) and SR intake/uptake Ca^2+^ channels parameters (a_SERCA_, a_RyR_), for a total of 17 parameters.

##### Parameters search space:

To reduce the parameter search space we used mRNA expression data to estimate conductivity ranges [57]. These mRNA derived baseline bounds were expanded based on additional experimental data. The bounds do not set values in the model but are used to infer a reduced space to search for model parameters. We used mRNA expression data to estimate protein density to provide bounds on the parameter search space. While the link between mRNA and function is not perfect, mRNA of surface membrane and SR transporters as well as Ca^2+^ buffers are correlated with protein density [78]. The total mRNA expression for a specific transporter was computed as the sum of the mRNA expression data of all the genes encoding that protein (Table 5).

**Table 5:** Ion channels and other proteins and relative genes in in the human fetal ventricular myocyte.

Channel/Protein	Genes	Channel/Protein	Genes
I_Na_	*SCN1A, SCN2A, SCN3A, SCN4A, SCN5A, SCN7A, SCN8A*	I_CaT_	*CACNA1G, CACNA1H, CACNA1I*
I_NaCa_	*SLC8A1, SLC8A2, SLC8A3*	I_pCa_	*ATP2B1, ATP2B2, ATP2B3, ATP2B4*
I_Ks_	*KCNQ1, KCNE1*	I_pK_	*KCNK1–6, 9, 10, 13,16–18*
I_Kr_	*KCNH2*	SERCA	*ATP2A1, ATP2A2, ATP2A3*
I_K1_	*KCNJ2, KCNJ4, KCNJ12*	RyR	*RYR2*
I_to_	*KCND3, KCNA4*	Troponin C	*TNNC1*
I_NaK_	*ATP1A1, ATP1A2, ATP1A3, ATP1A4, ATP1B1, ATP1B2, ATP1B3, ATP1B4*	Calsequestrin	*CASQ2*
I_CaL_	*CACNA1C, CACNA1F*	I_f_	*HCN1, HCN2, HCN3, HCN4*

##### mRNA data usage:

We aimed to account for age and the mix of tissue type when using available mRNA data. We first assumed that the proportion of mRNA per unit volume expressed between the atrium and the ventricle are the same in the fetus as in the adult and that, in case of mixed cardiac tissue, the samples are composed almost exclusively of atrial and ventricular cells so that the expression value for the mixed cardiac tissue is approximately the mean expression value for atrium and ventricle values.

These simple assumptions are needed to interpret the limited published mRNA databases. For each protein of interest, we can compute the ratio of the fetal value (FV) and adult value (AV) from Database 1 and Database 2. We then rescaled the value of each channel conductance in the TT2 model by FV/AV in order to obtain a prediction of the channel conductance in our fetal model. These estimates were not used as the final values in the model but were used to give initial bounds on each parameter in Table 6. In cases of uncertainty in protein density we took the conservative approach of increasing the bounds to cover the broadest range of potential values.

**Table 6:** Values for model parameters of membrane and intracellular proteins predicted at different gestational times from mRNA gene expression as described in text and for the TT2 model for adult as well as the Fabbri model for funny current and choice of boundaries values considered to restrain the parameter space.

Parameter	<10 gw [54]	13 gw [53]	TT2 (adult)	10 gw [56]	10 gw [57]	Search space
**g**_**Na**_	5.959	10.175	14.838			[5.959 14.383]
**k**_**NaCa**_	623.6	2841.1	1000		1471	[623.6 2841.1]
**g**_**Ks**_	0.112	0.159	0.392			[0.112 0.392]
**g**_**K1**_	–	3.083	5.405			[1 5.405]
**g**_**Kr**_	0.076	0.089	0.153			[0.076 0.153]
**g**_**to**_ (epi) (endo)	0.221 0.055	0.239 0.059	0.294 0.073			[0.055 0.294]
**p**_**NaK**_	1.504	0.643	2.742			[1.362 4.086]
**g**_**CaL**_	5.06e-2	5.11e-2	3.98e-2	0.5e-2		[0.5e-2 5.11e-2]
**g**_**pK**_	0.008	0.0044	0.0146			[0.0044 0.0146]
**g**_**pCa**_	0.245	0.09	0.1238			[0.09 0.245]
**g**_**bNa**_			2.9e-4			[1.45e-4 4.35e-4]
**g**_**bCa**_			5.92e-4			[2.96e-4 8.88e-4]
**a**_**TropC**_	0.16	0.31	1			[0.16 1]
**a**_**CASQ**_	0.2	0.46	1			[0.2 1]
**a**_**SERCA**_	0.3	0.45	1	0.3		[0.3 1]
**a**_**RyR**_	0.17	0.86	1			[0.17 1]
	(mix tissue)	(ventricle)	**Fabbri** **(SAN adult)**			
**g**_**fK**_	0.109	0.1773	0.2053			[0 0.1773]

Database 1 reports mRNA in samples of mixed atrial and ventricle tissue for adults but separate atrial and ventricle measurements for fetal preps. We assume that the proportion of ventricle mRNA AV (adult) and FV (fetal) for a given protein remains the same in mixed adult (A) and fetal (F) preparations (AV/FV = A/F), and that the mixed mRNA levels are the average of the corresponding atrial and ventricle expression levels, such that F=(FA + FV)/2. This allows us to calculate an estimate of the adult ventricle expression level, AV = 2A/ (1 + FA/FV).

Database 2 reports mixed fetal and adult expression and one adult ventricle result. We estimated mRNA expression values for the fetal ventricle as FV = AV x F/A where and A is the average of the two adult mixed tissues.

We used Database 3 to estimate the gestational age of fetal data in Database 2, comparing mRNA expression data in fetal ventricles at 9 and 16 gw. Assuming a linear increase/decrease of proteins density within this time window we extrapolated indicative values for 10 gw which placed Database 2 at an approximate gestational time prior 10 gw.

We used the two predicted values from Database 2 and 1 as bounds to initially constrain the parameter search space. In cases, where the channel conductance estimates prior to 10 gw, the estimate at 13 gw and the value for TT2 were not consistent with a linear change, we enlarged the parameter search space to cover all three values (Table 6).

In simulations, low values of the sodium-potassium pump conductance generated abnormal APs, and the model did not reach a limit cycle. We also found that mRNA expression levels were lower in fetuses, despite animals reporting a higher function of I_NaK_ in fetal than adult cardiac cells. We therefore increased the search range of I_NaK_ to ± 50% of the TT2 model. We adopted the same approach for both Na^+^ and Ca^2+^ background currents as the proteins involved in the generation of these currents are not defined.

Fetal L-type Ca^2+^ channel mRNA expression is higher compared to adult both in Database 1 and 2. This is in contrast to reports in human and animals studies that found that the L-type current is underdeveloped in fetal compared to adult so we expanded the conductance search space to include conductance values predicted from human fetal mRNA expression data at 10 gw [56] and we did the same for the sodium-calcium exchanger [57] and the SERCA protein [56]. For the funny current we started with a reference conductance value in adult SAN from the Loewe model. As we did not have specific SAN tissue samples, we used the mixed tissue expression levels in adults to generate bounds by scaling the reference conductance by F/A in Database 2 (where A and F correspond to mRNA expression in adult and fetal mixed tissue respectively) and by FV/A (being FV the mRNA expression in fetal ventricle) in Database 1. The final bounds are defined in Table 6.

##### Non-implausible fetal action potential and calcium transient: History matching:

We initially used Bayesian history matching technique [79–81] to restrict the parameter search space in order to characterise points in the input space that lead to a model with a simulated AP and Ca^2+^ transient which fall within the range of the experimental mean ± 3 STD, in accordance with the Pukelsheim three sigma rule [82].

Using the parameter space bounds (Table 6) we trained a Gaussian Process Emulator for: V_max_, RMP, APD at different voltages APD_0,_ APD_-20_, APD_-40_, APD_-60_ (AP); t50_up_, t_peak_, t25, t50, t75, t90 (Ca^2+^ transient) model outputs. In each History matching wave Emulators output were compared with experimental data using the following implausibility measure:


$$I_{j}^{\text{2}}(x):=\frac{{\left(E\left[f_{j}(x)\right]-E\left[Y_{j}\right]\right)}^{\text{2}}}{Var\left[f_{j}(x)\right]+Var\left[Y_{j}\right]}$$

for any feature *j* and for any point x, where E[*f*_*j*_(x)] is the emulator prediction with its variance Var[*f*_*j*_(x)] and E[*Y*_*j*_] is the experimental mean with its variance Var[*Y*_*j*_]. Points with an implausibility score above a chosen *cut-off* for some feature *j* are considered implausible

Iteration algorithm:

1. First wave: 300 equally distributed quasi-random points were selected using Sobol low-discrepancy-sequence (LDS) sampling from the initial parameter search space. Next waves: the previous wave non-implausible space was sampled with part-and-select algorithm (PSA) to obtain 300 simulator inputs.
2. The simulator was evaluated in the input points obtained from the previous step and points which produced abnormal simulation output (failed simulation, self-excitation or failure to repolarise) were excluded.
3. The remaining inputs and outputs together with simulations from the 3 previous waves (in order to maintain the same level of accuracy at the edge of the parameter non-implausible space border) were used as training dataset for emulators.
4. First wave: the emulators were evaluated on 500,000 points selected from the initial input space using a Latin hypercube sampling (LHS). Other waves: the emulators were evaluated in all points of the non-implausible space of the previous wave.
5. Implausibility was computed for each output feature, and the cut-off threshold applied to the maximum implausibility value over all features to determine if input points are non-implausible. If the number of non-implausible points was less than 100,000, new non-implausible points were generated using the clouding technique described in [79].
6. We repeated this algorithm reducing the cut-off threshold, of same step-by-step amount as in [79], until the cut-off value = 3 (because of the three sigma rule) and the non-implausible space was 95% or more of the input space for the current wave.

For a complete description of history matching technique and GPEs construction see [83].

##### Best fit search:

History matching generates a range of non-implausible parameter values. However, for many applications a single cell model parameter set is desirable. To determine a single best parameter set following the history matching approach we used GPEs in place of the simulator to further constrain the parameter space, by incrementally reducing the cut-off threshold. This was done in steps in order to ensure model stability and coherence around the final parameter choice. The algorithm is the same as described above but this time we used as initial input space the final non-implausible space obtained from history matching and, at any step, only points whose emulator outcome distance from the experimental mean was below a specific degree of uncertainty were selected to constitute the new input space for next step. We started with an uncertainty of 3 STD and reduced this until we reached 1 SEM or no points were found anymore. The difference with history matching implausibility measure is that we are now neglecting the numerical uncertainty of the emulators prediction, based on the assumption that emulators have already reached a high level of accuracy. From the last set of points predicted by the emulators to be in the smallest range possible of at least 1 SEM from the experimental mean, 300 points were selected and among them the input point whose simulator output was closest to experimental mean was chosen as the best fit.

### Global sensitivity analysis

Global sensitivity analysis (GSA) was performed using a variance-based Sobol (Saltelli) method to quantify the relative influence of ionic and Ca^2+^ -handling parameters on action potential and Ca^2+^-transient features. GPEs were used in place of the full simulator. Each conductance and Ca^2+^-handling parameter was varied independently by ±30% around its calibrated value. Parameter sets were generated using Saltelli sampling, and first- and total-order Sobol indices were computed to assess direct and interaction effects on model outputs. GSA was performed for both the hfVM and TT2 models to enable comparison of fetal and adult electrophysiological control mechanisms. The GSA was implemented using GPErks (https://github.com/stelong/GPErks).

### Numerical methods

T-type current fit was performed in Matlab using *lsqcurvefit* (method: nonlinear least square, algorithm: trust-region-reflective, initial guess: t_0_ = 0). Model simulations were run with Julia Language using ODE solver CVODE_BDF (CVode Backward Differentiation Formula) with maximum time step of 0.5 ms and model outputs were considered after a 2000 time periods adjustment in order to allow the model to reach its stability. The cell model is available through the CellML repository (https://models.physiomeproject.org/workspace/d90). GPEs training, history matching, best fit search and GSA were performed in Python [84].

## Results

All simulations are shown after stimulating the model for 2000 beats to ensure the cell model has reached a limit cycle.

### Calibration of T-type calcium channel

Ca^2+^ current measurements were fitted using either T-type or a combination of T-type and L-type currents. Fitted current traces that accounted for both L-type and T-type Ca^2+^ channels had a smaller error, especially in the case of cell 2–5, whose T-type Ca^2+^ response is more affected by L-type current interference. The best fit across all cells was obtained using the Dokos T-type model for cell 1 and Demir model for cell 2–5, in combination with the Dokos L-type model in all cases. Specifically, cell 1 shows different behaviour than the other four cells, in that the Ca^2+^ current reaches its reversal potential at a voltage higher than 50 mV while other cells currents reach it at voltages close to 40 mV. The current on time traces of cell 1 are characterised by a smaller time of decay at negative voltages and a longer decay at positive voltages than the current of cells 2–5. Cell 1 has a higher peak current of 25 pA/pF, compared to the other cells where the maximum current occurs at approximately 10 pA/pF. These characteristics of cell 1 resemble those of the T-type Dokos model. In contrast cell 2–5 behaviour is more similar to the Demir T-type model (see S2 Text). For this reason it was decided to consider at this point two different models for the T-type Ca^2+^ current: Model 1, which uses T-type current equations from Dokos and T-type conductance equal to 0.95 nS/pF obtained from the fitting of cell 1 using the combination T-type Dokos + L- type Dokos models, and Model 2, with T-type equations from Demir and conductance of 0.2 nS/pF obtained as the average of T-type conductance of cell 2–5 fitted with the combination T-type Demir + L-type Dokos models.

**Fig 1:**
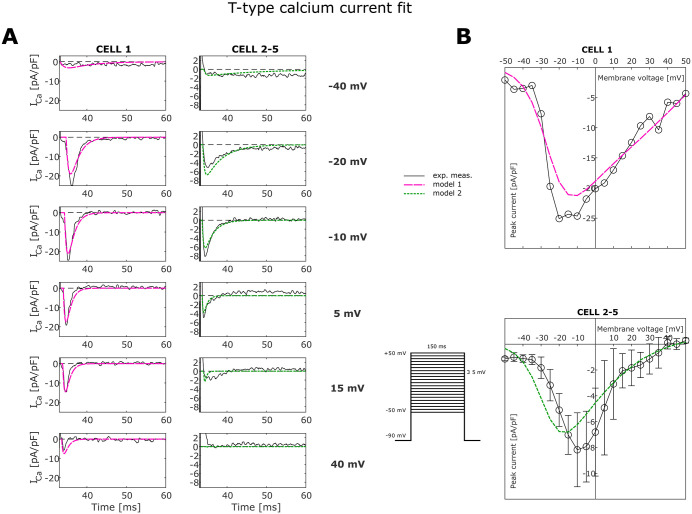
T-Type calcium current fit. **(A)** In the left column the experimental traces from CELL 1 cleared of L-type current are compared with Dokos T-type model with imposed conductance (Model 1) while in the right column the average of cleared experimental traces from CELL 2 to 5 are compared with Demir T-type model with imposed conductance (Model 2). **(B)** Same comparison as in A between peak current on voltage curves. The average peak current curve at the bottom is plotted with its standard deviation bars.

Fig 1 shows the best fitted T-type model for each group of cells after subtraction of simulated L-type current interference from the fit with Dokos L-type model. The peak current curve (panel B) for cell 1 shows a very good agreement between the model and experimental data, with the model matching both the reversal potential value and the peak voltage. Cell 2–5 simulated peak current curves show a good correspondence with experimental data for the reversal potential. The simulated peak current is more negative than the real one for negative voltages. This may be due to fitting only the channel density, while the gating variables are taken from the original model, or that there was a greater impact of L-type channel on the cell 2–5 traces than on cell 1. The T-type channel model is based on experimental measurements obtained at 13.5 gw. The conductance was scaled to estimate the T-type channel density at 10 gw (fetal age represented by the hfVM model), giving a value of 1.587 nS/pF for Model 1 and 0.334 nS/pF for Model 2. We did not use the fitted L-Type Ca^2+^ model as the L-type channel was partially inhibited by nifedipine during the experiments.

### Calibration based on action potential and calcium transient

We created two forms of the hfVM, each with one of the two proposed T-type models. We estimated the remaining parameters for both cases. The fit of fetal AP and Ca^2+^ transient obtained a better agreement using model 1 for the T-type channel, while with model 2 for the T-type channel the whole cell model stopped converging after fewer iteration waves and at a larger uncertainty distance from the experimental mean. Therefore, we used T-type model 1 for the final version of the hfVM and hereinafter present only the results obtained with this version.

#### History matching.

**Fig 2:**
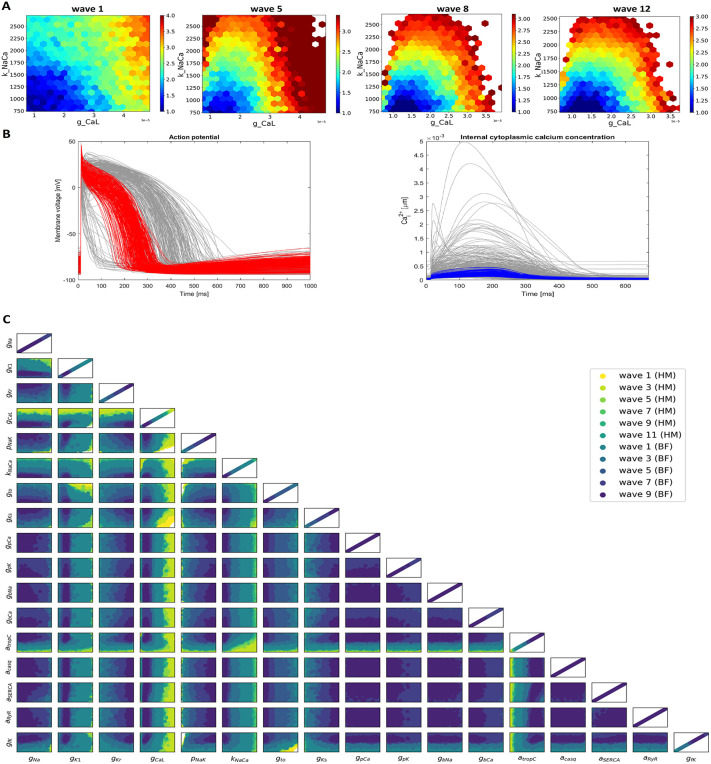
Model fitting procedure. **(A)** Progressive reduction of the parameter search space in the plane G_CaL_ - K_NaCa_ for waves 1, 5, 8 and 12 (last wave) of history matching. Coloured points are the non-implausible points for the corresponding wave, i.e., points where the implausibility measure is lower than the cut-off considered (implausibility measure being higher for red points and lower for blue ones), while the white area represents points deemed to be implausible. **(B)** Grey lines represent AP (left) and Ca^2+^ transient (right) each obtained as output of 300 input points selected with LDS sampling in the initial parameter search space, while the coloured line are the AP (red) and Ca^2+^ transient (blue) outputs of the non-implausible points from the last wave of history matching. **(C)** The parameter space was progressively reduced, first using history matching and subsequently refined using the best-fit procedure described in the text. The last wave (darkest blue) corresponds to the points predicted by the GPEs to be within 2 SEM distance from experimental mean, from which we computed the best fit.

Fig 2C shows the progressive reduction of the non-implausible region of each history matching and best fit search wave within the parameters input space. In Fig 2A an example of the input space reduction in the plane G_CaL_ - K_NaCa_ obtained with the history matching technique is shown for selected waves. The colour of each hexagonal pixel corresponds to the point with minimum implausibility measure within the pixel space. The white area of each wave plot is the space portion already found by the previous wave to be implausible so not included in the present wave input space.

**Fig 3:**
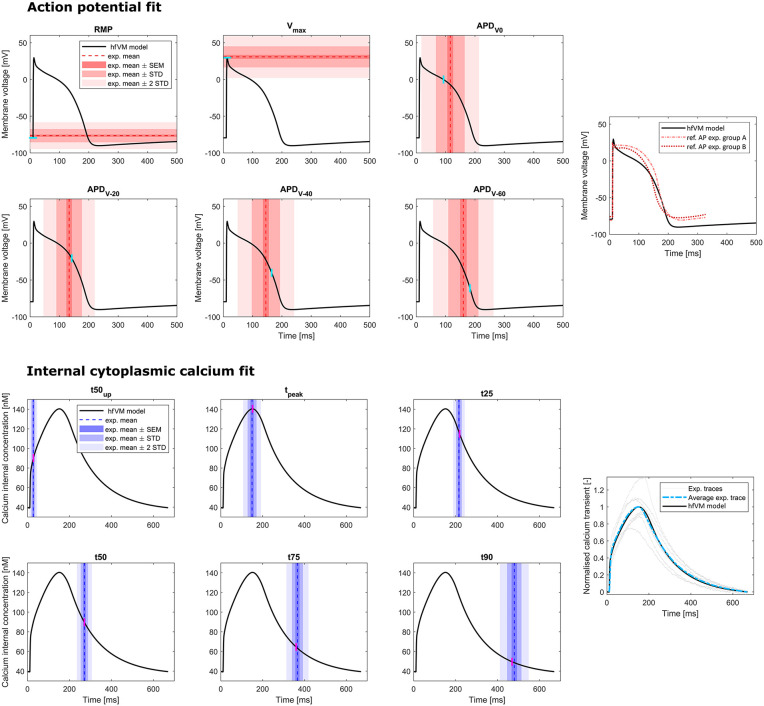
Final results of the fit showing fetal AP (1 Hz) and Ca^2+^ transient (1.5 Hz) features in comparison between the hfVM model and the experimental measurements that were used to calibrate it. In the right boxes we plot AP (above) generated by the model against reference AP as reported in [30], and the simulated Ca^2+^ transient (below) against the experimental Ca^2+^ transient curves recorded from different cells and their average trace.

The results of the history matching, in terms of non-implausible AP and Ca^2+^ transient simulations, are shown in Fig 2B. Grey curves correspond to 300 parameter combinations sampled randomly from the initial input space (Table 6). Coloured curves show model outputs for the parameter sets remaining after selection using history matching. These represent non-implausible configurations of AP and Ca^2+^ transient of the ventricular myocyte of a human fetus at approximately 10 gw in accordance with the experimental measures considered in this study. Further progressive reduction of the parameter search space from the final non-implausible region obtained with history matching (see above), identified a final best fit model, where comparisons of the experimental data and the best model are presented in Fig 3.

### Validation

**Fig 4:**
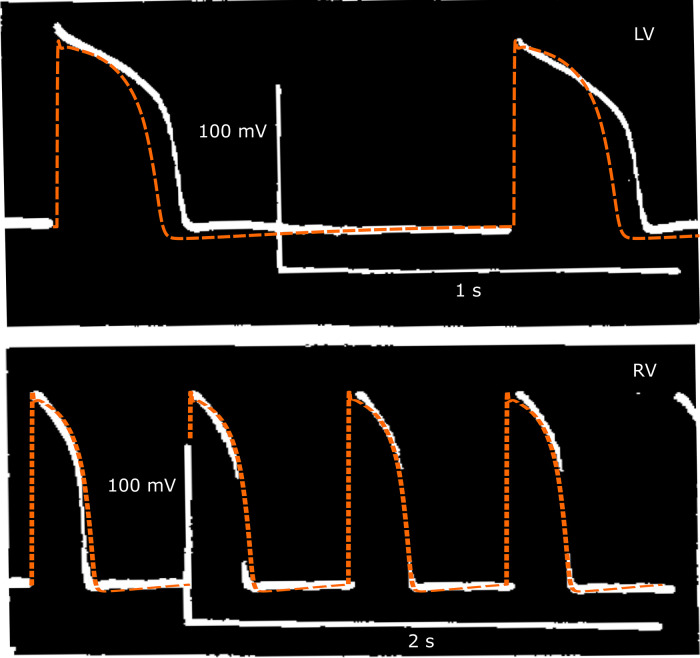
AP recordings from left (above) and right (below) ventricular myocytes of human fetal heart at 7-12 gw as reported in Study 2 [51] (white) and comparison with AP simulated with the hfVM model (orange) adapted for cardiac time period, external ionic concentrations and absolute temperature to match experimental conditions and beating frequency in each of the two cases.

Validation against Study 2 shows that the model AP features are all in the experimental range except for the maximum value (V_max_) is slightly high (Table 7). This might be expected, as the target V_max_ value use for calibration was outside the range reported in Study 2 and potentially reflects variation in experimental measurements. Looking at AP recordings shown in Fig 4 we can see that experimental AP exhibits hyperpolarisation at the end of phase 3, which is captured by the hfVM model. For both cells, simulated and experimental AP are in good agreement, especially for the right ventricle cell whose AP is resembled very closely by the fetal model.

**Table 7:** AP validation: comparison of characteristic features of AP simulated using the hfVM model against AP experimental data of human fetal ventricle reported in Study 2 [51].

	Experimental		hfVM model	In range
	Range Mean			
**RMP [mV]**	-70 - -85		-78.20	yes
**V**_**max**_ **[mV]**	18 - 30	25	30.22	no
**V**_**amp**_ **[mV]**	95 - 120	110	108.42	yes
**APD95 [ms]**	185 - 310		260	yes

**Fig 5:**
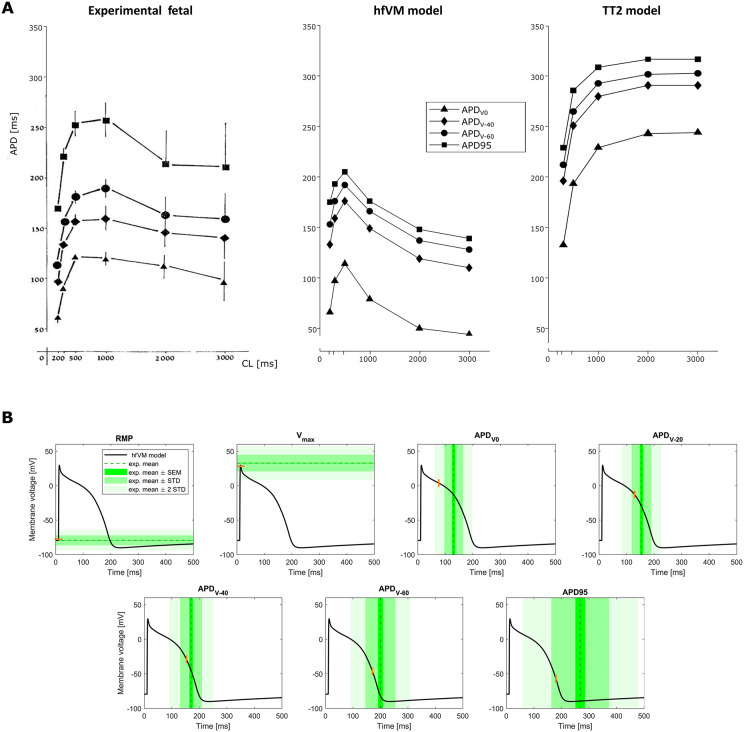
Validation of model AP against experimental measurements data from Study 3 [52]. **(A)** Action potential duration (APD) at given voltage levels (0, -40, -60 mV) and at 95% repolarisation is shown for different cycle lengths (200, 300, 500, 1000, 2000 and 3000 ms) for fetal experimental measurements, the hfVM model and the TT2 adult model. **(B)** AP (1 Hz) features comparison between the hfVM model and the experimental measurements from fetal ventricles as reported in the study.

Fig 4 shows validation of the model against experimental data reported in Study 3 [52]. APD at different voltage values are plotted against cardiac cycle length and we compare fetal experimental data, the hfVM fetal model and the TT2 model for adult (panel A). We show that, unlike the adult ventricular myocyte whose APD typical behaviour is represented here by TT2, the fetal ventricular myocyte APD tends to increase with increasing cycle length (frequency decreasing) until a certain point, after which APD starts decreasing. This behaviour, which appears to be a distinctive characteristic of the human fetal heart compared to adult, is captured by the hfVM model. The APD versus cycle length curve reaches its maximum value at 500 ms (2 Hz) for the model while in the experiments the maximum corresponds to 1000 ms (1 Hz). Simulated APD values are smaller than the experimental ones, but this is likely due to the fact that the experimental APs used for calibrating the model exhibit shorter APD than Study 3. However, when comparing predicted APD values against AP measurements from Study 3, all predicted features are still within 1–2 standard deviations from the experimental mean (Fig 5B).

**Fig 6:**
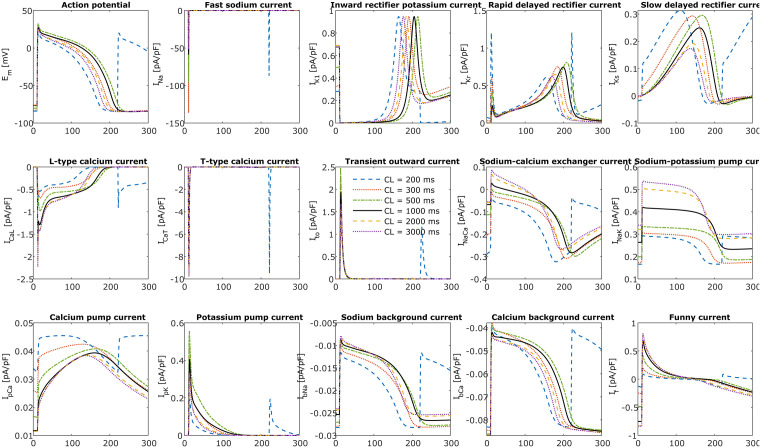
Simulation membrane ionic currents for the fetal myocyte computed using the hfVM model with different stimulation periods corresponding to different cycle lengths (200 ms, 300 ms, 500 ms, 1000 ms, 2000 ms, 3000 ms).

Fig 6 shows the simulated ionic currents at the pacing cycle lengths (200, 300, 500, 1000, 2000 and 3000 ms) in Fig 5. The biphasic change in APD in Fig 5 can be attributed to an initial prolonging of the action potential when the cycle length increases from 200 to 500ms as the cell becomes less refractory. This is seen as an increase in the fast sodium channel current. As the pacing cycle length increases from 500 to 3000ms, the funny current increases, depolarising the resting membrane potential, causing an attenuation of the fast sodium channel. We also see a decrease in the sodium concentration causing the sodium calcium exchanger to operate in reverse mode during the plateau phase at longer pacing cycle lengths, this causes the shortening of the APD. Setting the funny current conductivity to zero removes the APD shortening and leads to a more conventional plateau of the APD at longer cycle lengths.

**Fig 7:**
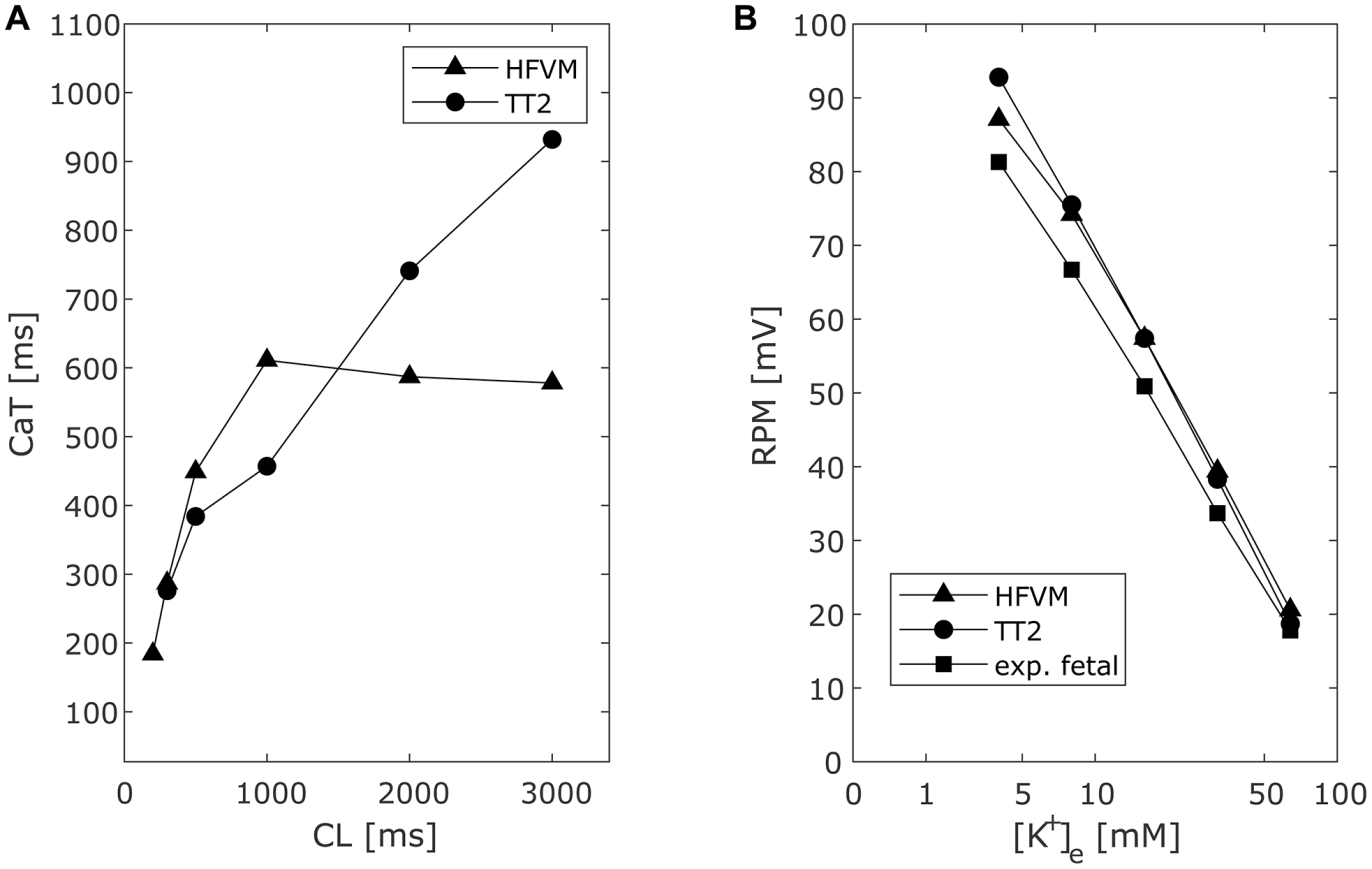
Comparison of Ca^2+^ transient (CaT) duration in human fetal and adult ventricular myocyte models and extracellular K^+^ -dependent resting membrane potential between human fetal and adult  ventricular myocyte models and experimental data. A. Ca^2+^ transient duration measured as 95% decreasing from peak computed at different pacing frequencies and correspondent cycle length for hfVM e TT2 models. B: AP resting value variation with varying external K^+^ concentration for hfVM, TT2 and data recorded from human fetal ventricular myocytes at 17-24 gw reported in literature [85].

Like the TT2 model AP, the hfVM model AP resting membrane potential decreases when increasing external K^+^ concentration (Fig 7B). Ca^2+^ transient duration in the fetal model decreases with decreasing external Ca^2+^ concentration and increases with increasing cycle length until 1000 ms, after which it decreases as cycle length increases (Fig 7A). This is in contrast with the behaviour of the adult cardiac myocyte predicted by the TT2 model, and it could indicate a characteristic feature of the fetal heart consistent with the more complex APD dependence on pacing frequency.

### Global sensitivity analysis

**Fig 8:**
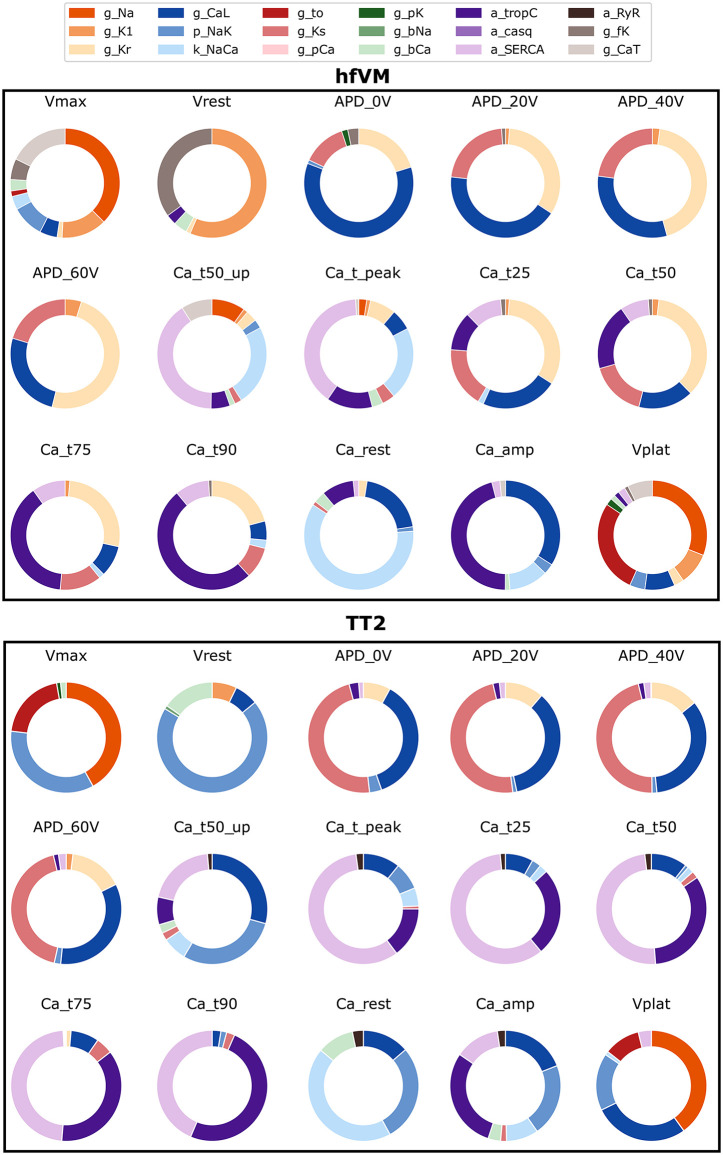
Global sensitivity analysis (total variance) of model parameters for characteristic features of simulated AP and Ca^2+^ transient for both hfVM model with stimulus period of 353 ms and TT2 models with stimulus period of 1000 ms.

Global sensitivity analysis was performed on the fitted cell model parameters for AP and Ca^2+^ transient features. Fig 8 shows that the maximum voltage in the hfVM is mostly influenced by I_Na_ current, as in TT2, but also by I_CaT_ current while in the TT2 model, the I_CaT_ contribution is covered by the I_NaK_ and I_to_ currents. The main currents responsible for the resting membrane potential in hfVM are I_K1_ and the funny current, while in the TT2 model RMP is mostly determined by I_NaK_. It is interesting to notice that even if the p_NaK_ value is higher in hfVM than TT2, the sodium-potassium pump has a diminished effect on AP and Ca^2+^ transient of the fetal model, with its contribution seemingly replaced by other currents. APD is influenced almost entirely by I_Kr_, I_Ks_ and I_CaL_ in the hfVM model as well as in TT2, but while in the adult model the major contribution is played by I_Ks_ in the fetal model the relative importance of the two potassium channels appears reversed, with I_Kr_ having the predominant effect on APD in the fetal model. I_Kr_ is also largely responsible for Ca^2+^ transient duration, while in TT2 its impact on cytosolic Ca^2+^ concentration is limited. In general, we see a reduced influence of the L-type Ca^2+^ current, consistent with findings of a reduced contribution of this current in the fetal heart cell compared to the adult.

### hfVM model

**Fig 9:**
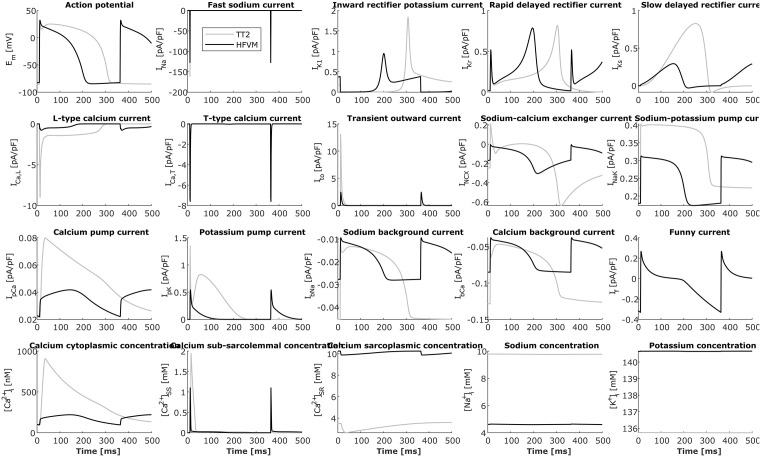
Simulation membrane ionic currents and ionic internal concentrations for the fetal myocyte computed using the hfVM model in comparison with those compuited for the adult cell using the TT2 model.

The final fetal model has the same value for external concentrations and absolute temperature as in TT2, and a cycle length of 353 ms corresponding to a 10 gw fetal heart beating at 170 bpm. In Fig 9 all hfVM model currents and internal ionic concentrations are shown in comparison with the TT2 model for the adult. In Table 8 characteristics of AP and Ca^2+^ transient morphology are compared between the hfVM model and the TT2 model for the adult. The final set of parameters obtained through model calibration is listed in Table 9.

**Table 8:** Characteristics of simulated AP and Ca^2+^ transient morphology are compared between the hfVM model (fetal) and the TT2 model (adult).

	hfVM	TT2
**Time period** [ms]	353	1000
**RMP** [mV]	-82.56	-85.42
**V**_**max**_ [mV]	32.06	32.49
**V**_**amp**_ [mV]	114.63	117.91
**APD** [ms]	200	308
**Ca**_**rest**_ [nM]	100.05	104.65
**Ca**_**max**_ [nM]	220.86	907.17
**Ca**_**amp**_ [nM]	120.81	802.51
**CaD** [ms]	326	457

**Table 9:** Values of parameters in the hfVM model obtained from the fitting of the model compared to their corresponding value in the TT2 model.

Parameter	hfVM	TT2	Parameter	hfVM	TT2
**g**_**Na**_ [nS/pF]	7.627	1.484e1	**g**_**fK**_ [nS/pF]	1.126e-1	0
**g**_**K1**_ [nS/pF]	2.785	5.405	**g**_**pK**_ [nS/pF]	5.949e-3	1.460e-2
**g**_**Kr**_ [nS/pF]	1.515e-1	1.530e-1	**g**_**pCa**_ [nS/pF]	1.361e-1	1.238e-1
**g**_**Ks**_ [nS/pF]	3.752e-1	3.920e-1	**g**_**bNa**_ [nS/pF]	1.598e-4	2.900e-4
**g**_**CaL**_[L/Fs]	1.087e-2	3.980e-2	**g**_**bCa**_ [nS/pF]	3.959e-4	5.920e-4
**g**_**CaT**_ [nS/pF]	1.587	0	**a**_**TropC**_ [-]	8.678e-1	1
**k**_**NaCa**_ [pA/pF]	6.814e2	1.000e3	**a**_**CASQ**_ [-]	5.422e-1	1
**p**_**NaK**_ [pA/pF]	3.998	2.724	**a**_**SERCA**_ [-]	9.941e-1	1
**g**_**to**_ [nS/pF]	5.719e-2	2.940e-1	**a**_**RyR**_ [-]	2.387e-1	1

## Discussion

We have performed a comprehensive review of fetal electrophysiology data and used this to create and extensively validate the first biophysical model of the hfVM. We have shown how mRNA, ion channel, AP and Ca^2+^ transient data can be used to constrain a model. We have calibrated a deterministic and an uncertain model and we have performed the first GSA comparing the relative contribution of different ion channels to emergent AP and Ca^2+^ transient features in fetal and adult myocyte models.

The AP morphology of the hfVM model is in agreement with AP data of human fetal ventricular myocytes aged 7–12 gw, exhibits a shorter APD, a more positive RMP than the adult ventricular myocyte, and hyperpolarisation at the end of phase 3 of the cardiac cycle [51], due to the presence of funny current in the fetal ventricular myocyte which is absent in the adult ventricular myocyte. The APD is shorter at lower pacing frequencies in accordance with the behaviour of human fetal ventricular myocytes in the experiments, while in adult myocytes APD tends to increase monotonically as the frequency decreases. The hfVM model AP resting membrane potential decreases when increasing external K^+^ concentration in accordance with experimental data from human fetal ventricular myocytes [85].

We found that despite NaK pump conductance being higher in the fetal than the adult cell model, its significant contribution to the RMP in the adult cell is diminished in the fetal cell, and thus the AP resting value in the hfVM model depends mostly on the funny current and I_K1_. The major contribution of I_K1_ to the RMP has been observed in models of neonatal rat ventricle [86]. The repolarisation time of the hfVM model AP depends predominantly on I_Kr_ while in TT2 it is mainly influenced by I_Ks_, although this difference may reflect the specific calibration of the TT2 model [87]. A predominant role of I_Kr_ compared to I_Ks_ in the APD is consistent with a model for the neonatal rat [86], also based on TT2 model, and modelled human-induced pluripotent stem cells (hiPSC)-derived cardiomyocytes [88].

The Ca^2+^ transient of the fetal model has a similar morphology to the one measured in fetal rats [46], both for shape and amplitude. Compared to the adult, the fetal Ca^2+^ transient curve is much less steep prior to its peak. This is consistent with the entry of Ca^2+^ into the fetal heart cell via T-type Ca^2+^ channels and the sodium-calcium exchanger (operating in reverse mode), rather than via L-type channels inducing Ca^2+^ release from the SR as occurs in adults. The limited contribution in early gestational age of the SR in releasing Ca^2+^ in the cytoplasmic intracellular space is responsible for the lower Ca^2+^ amplitude in the fetus than the adult. This is highlighted in the GSA results where we can see that the contribution of I_CaL_ to the Ca^2+^ concentration initial rising time is limited in contrast to the adult model, with I_CaT_ and I_NaCa_ playing this role instead. GSA results predicts that SR Ca^2+^ release plays a significant role in the rise of internal Ca^2+^ in fetal myocytes, although this is attenuated compared to the adult model, findings which are consistent with neonatal rat studies [86]. Ca^2+^ transient duration decreases while reducing external Ca^2+^ concentration, this behaviour has also been observed experimentally in human fetal ventricular myocytes [89].

The hfVM model simulated AP is in close approximation with both experimental [90,91] and simulated [88] ventricular-like hiPSC-derived cardiomyocytes. This indicates that those cells could be used experimentally to deduce ventricular cardiomyocyte behaviour and properties of the human early fetal heart. In the same way, Ca^2+^ transient in the model as well as the experimental Ca^2+^ current recordings from human fetal ventricles used for model calibration, resemble Ca^2+^ transient curves recorded in hiPSC-derived cardiomyocytes [91,92].

We fitted the hfVM model using Bayesian history matching approach, this approach has been adopted from engineering applications and is now being applied in biomedical applications. Alternative methods have been proposed for fitting cardiac models, including population [93] of models and translation approaches [94]. In contrast to populations of model approaches, history matching makes use of emulators, this reduces the computational cost and increases sample efficiency compared. Translation approaches where a validated model for one species, or in this case age, is translated to another, implicitly assume common ion channels in both systems. However, the absence of T-type Ca^2+^ channels in adult myocytes in adult myocytes would necessitate a modification of this method to translate an adult cell model to a fetal model.

Several groups have modelled immature cardiac electrophysiology, and our work complements and extends these efforts to human fetal ventricular physiology. Neonatal rodent [86] and hiPSC-CM frameworks [88,92] similarly emphasise reduced I_K1_, stronger reliance on sarcolemmal Ca^2+^ entry, and a greater contribution of I_Kr_ to repolarisation, broadly consistent with our findings. However, these studies typically reflect postnatal rodent or developmentally heterogeneous human hiPSC phenotypes rather than a defined gestational stage and therefore do not reproduce key fetal-specific features we identify, including the role of I_f_ and I_CaT_ with diminished I_K1_ in setting resting potential, and the dominance of I_Kr_ over I_Ks_.

We have created a framework for simulating fetal ventricular myocyte electrophysiology and Ca^2+^ dynamics. Limitations in data availability or completeness required certain assumptions to interpret or extrapolate data. Where possible, we have mitigated the impact of these assumptions on our final results by using these measurements to define prior parameter ranges for subsequent Bayesian fitting. In particular, we have had to estimate or extrapolate cell structure, volumes and capacitance values. These represent areas where additional systematic measurements would benefit model creation.

### Limitations

The use of mRNA gene expression data as a measure of protein density on the membrane surface is an approach that has been previously made in electrophysiological modelling [95–99], but it is recognised that this only provides an estimation of protein function [100]. Calibration of the model for Ca^2+^ features was based on Ca^2+^ transient recordings from human fetal cells aged 12–17 gw, while AP data for calibration come from fetal myocytes aged 10 gw, which was also the fetal age represented by the hfVM model. The experimental APs from study 2 used for the validation of the hfVM model AP were recorded at room temperature, while the hfVM has been adapted to room temperature only for the absolute temperature, while the channel dynamics parameters are taken from the TT2 model which was calibrated at body temperature. Human fetal data are inherently scarce. The proposed model represents the best estimates from the available data, but there are still areas of high uncertainty. The model we have proposed provides a framework that integrates the available data in a common framework, providing a quantitative basis for interpreting new data and testing hypotheses.

## Conclusion

In this study we present the first model of human fetal ventricular myocyte electrophysiology which can replicate key features of AP depolarisation, repolarisation and Ca^2+^ transient dynamics of the fetal cardiac cell at 10 gw. The hfVM model was developed using calibration data and subsequently validated using data from human fetal ventricular myocytes at early gestation. This model provides an important tool for investigating fetal arrhythmias, other gestational cardiac conditions, and for advancing the understanding of human fetal heart development. This hfVM model has the potential to serve as a basis for future models of the human fetal myocytes throughout gestation as well as in the neonatal period.

## Supporting information

S1 Text HFVM model equations and parameters. (PDF)

S2 Text Human fetal T-type calcium current. (PDF)
