# Supplementary material for: A model for the human fetal ventricular myocyte electrophysiology
Source: PLoS Comput Biol. 2026 Jan 27;22(1):e1013889. doi: 10.1371/journal.pcbi.1013889 (PMC12863696; doi:10.1371/journal.pcbi.1013889)
Supplement: S1 Text — (PDF) [file pcbi.1013889.s001.pdf]

HFVM model equations and parameters

Contents

Model equations 2

- Membrane potential . . . 2
- Nernst potentials . . . 2
- Fast sodium current . . . 2
- Inward rectifier potassium current . . . 3
- Rapid delayed rectifier potassium current . . . 3
- Slow delayed rectifier potassium current . . . 4
- L-type calcium current . . . 4
- T-type calcium current . . . 5
- Sodium-calcium exchanger current . . . 5
- Sodium-potassium pump current . . . 5
- Transient outward potassium current . . . 5
- Funny current . . . 6
- Calcium pump current . . . 6
- Potassium pump current . . . 6
- Sodium background current . . . 6
- Calcium background current . . . 6
- Calcium concentration . . . 6
- Sodium concentration . . . 7
- Potassium concentration . . . 7

Model parameters 8

- Physical constants . . . 8
- Cell geometry . . . 8
- ODEs initial conditions . . . 8
- Stimulus . . . 9
- Calcium dynamics . . . 9
- Ion external concentrations . . . 9
- Other parameters . . . 9
- Ion channels and transporters conductance . . . 9

Model equations

Membrane potential

$$I_{ion} = I_{Na} + I_{K1} + I_{Kr} + I_{Ks} + I_{CaL} + I_{CaT} + I_{to} + I_{NaK} + I_{pK} + I_{pCa} + I_{bNa} + I_{bCa} + I_f$$

$$\frac{\mathrm{d}V}{\mathrm{d}t} = -(I_{ion} - I_{stim})$$

Nernst potentials

$$E_{Na} = \frac{R \cdot T}{F} \cdot \ln\left(\frac{[Na]_e}{[Na]_i}\right)$$

$$E_K = \frac{R \cdot T}{F} \cdot \ln\left(\frac{[K]_e}{[K]_i}\right)$$

$$E_{Ks} = \frac{R \cdot T}{F} \cdot \ln\left(\frac{[K]_e + P_{KNa}[Na]_e}{K]_i + P_{KNa}[Na]_i}\right)$$

$$E_{Ca} = \frac{R \cdot T}{2 \cdot F} \cdot \ln\left(\frac{[Ca]_e}{[Ca]_i}\right)$$

Fast sodium current

$$m_{inf} = \frac{1}{\left(1 + exp\left(\frac{-56.86 - V}{9.03}\right)\right)^2}$$

$$\alpha_m = \frac{1}{1 + exp\left(\frac{-60 - V}{5}\right)}$$

$$\beta_m = \frac{0.1}{1 + exp\left(\frac{V + 35}{5}\right)} + \frac{0.1}{1 + exp\left(\frac{V - 50}{200}\right)}$$

$$\tau_m = \alpha_m \cdot \beta_m$$

$$\frac{\mathrm{d}m}{\mathrm{d}t} = \frac{m_{inf} - m}{\tau_m}$$

$$h_{inf} = \frac{1}{\left(1 + exp\left(\frac{V + 71.55}{7.43}\right)\right)^2}$$

$$\alpha_h = \begin{cases} 0.057 \cdot exp\left(\frac{-(V + 80)}{6.8}\right), & V < -40\,mV \\ 0, & \text{otherwise} \end{cases}$$

$$\beta_h = \begin{cases} 2.7 \cdot exp(0.079 \cdot V) + 310000 \cdot exp(0.3485 \cdot V), & V < -40\,mV \\ \frac{0.77}{0.13 \cdot \left(1 + exp\left(\frac{V + 10.66}{-11.1}\right)\right)} & \text{otherwise} \end{cases}$$

$$\tau_h = \frac{1}{\alpha_h + \beta_h}$$

$$\frac{\mathrm{d}h}{\mathrm{d}t} = \frac{h_{inf} - h}{\tau_h}$$

$$j_{inf} = \frac{1}{\left(1 = +exp\left(\frac{V + 71.55}{7.43}\right)\right)^2}$$

$$\alpha_j = \begin{cases} \frac{(-25428 \cdot exp(0.2444 \cdot V) - 6.948 \times 10^{-6} \cdot exp(-0.04391 \cdot V))(V + 37.78)}{1 + exp(0.311 \cdot (V + 79.23))}, & V < -40\,mV \\ 0, & \text{otherwise} \end{cases}$$

$$\beta_j = \begin{cases} \dfrac{0.02424 \cdot exp(-0.01052 \cdot V)}{1 + exp(-0.1378 \cdot (V + 40.14))}, & V < -40\,mV \\ \dfrac{0.6 \cdot exp(0.057 \cdot V)}{1 + exp(-0.1 \cdot (V + 32))}, & \text{otherwise} \end{cases}$$

$$\tau_j = \frac{1}{\alpha_j + \beta_j}$$

$$\frac{\mathrm{d}j}{\mathrm{d}t} = \frac{j_{inf} - j}{\tau_j}$$

$$I_{Na} = g_{Na} \cdot m^3 \cdot h \cdot j \cdot (V - E_{Na})$$

Inward rectifier potassium current

$$\alpha_{K1} = \frac{0.1}{1 + exp(0.06(V - E_K - 200))}$$

$$\beta_{K1} = \frac{3 \cdot exp(0.0002(V - E_K + 100)) + exp(0.1(V - E_K - 10))}{1 + exp(-0.5(V - E_K))}$$

$$xK1_{inf} = \frac{\alpha_{K1}}{\alpha_{K1} + \beta_{K1}}$$

$$I_{K1} = g_{K1} \cdot xK1_{inf} \cdot \sqrt{\frac{[K]_e}{5.4}} \cdot (V - E_K)$$

Rapid delayed rectifier potassium current

$$xr1_{inf} = \frac{1}{1d + exp\left(\dfrac{-26 - V}{7}\right)}$$

$$\alpha_{xr1} = \frac{450}{1 + exp\left(\dfrac{-45 - V}{10}\right)}$$

$$\beta_{xr1} = \frac{6}{1 + exp\left(\dfrac{V + 30}{11.5}\right)}$$

$$\tau_{xr1} = \alpha_{xr1} \cdot \beta_{xr1}$$

$$\frac{\mathrm{d}Xr1}{\mathrm{d}t} = \frac{xr1_{inf} - Xr1}{\tau_{xr1}}$$

$$xr2_{inf} = \frac{1}{1 + exp\left(\dfrac{V + 88}{24}\right)}$$

$$\alpha_{xr2} = \frac{3}{1 + exp\left(\dfrac{-60 - V}{20}\right)}$$

$$\beta_{xr2} = \frac{1.12}{1 + exp\left(\dfrac{V - 60}{20}\right)}$$

$$\tau_{xr2} = \alpha_{xr2} \cdot \beta_{xr2}$$

$$\frac{\mathrm{d}Xr2}{\mathrm{d}t} = \frac{xr2_{inf} - Xr2}{\tau_{xr2}}$$

$$I_{Kr} = g_{Kr} \cdot \sqrt{\frac{[K]_e}{5.4}} \cdot Xr1 \cdot Xr2 \cdot (V - E_K)$$

Slow delayed rectifier potassium current

$$xs_{inf} = \frac{1}{1 + exp\Big(\dfrac{-5 - V}{14}\Big)}$$

$$\alpha_{xs} = \frac{1400}{\sqrt{1 + exp\Big(\dfrac{5 - V}{6}\Big)}}$$

$$\beta_{xs} = \frac{1}{1 + exp\Big(\dfrac{V - 35}{15}\Big)}$$

$$\tau_{xs} = \alpha_{xs} \cdot \beta_{xs} + 80$$

$$\frac{\mathrm{d}Xs}{\mathrm{d}t} = \frac{xs_{inf} - Xs}{\tau_{xs}}$$

$$I_{Ks} = g_{Ks} \cdot Xs^2 \cdot (V - E_{Ks})$$

L-type calcium current

$$d_{inf} = \frac{1}{1 + exp\Big(\dfrac{-8 - V}{7.5}\Big)}$$

$$\alpha_d = \frac{1.4}{1 + exp\Big(\dfrac{-35 - V}{13}\Big)} + 0.25$$

$$\beta_d = \frac{1.4}{1 + exp\Big(\dfrac{V + 5}{5}\Big)}$$

$$\gamma_d = \frac{1}{1 + exp\Big(\dfrac{50 - V}{20}\Big)}$$

$$\tau_d = \alpha_d \cdot \beta_d + \gamma_d$$

$$\frac{\mathrm{d}d}{\mathrm{d}t} = \frac{d_{inf} - d}{\tau_d}$$

$$f_{inf} = \frac{1}{1 + exp\Big(\dfrac{V + 20}{7}\Big)}$$

$$\tau_f = 1102.5 \cdot exp\Big(\frac{-(V + 27)^2}{225}\Big) + \frac{200}{1 + exp\Big(\dfrac{13 - V}{10}\Big)} + \frac{180}{1 + exp\Big(\dfrac{V + 30}{10}\Big)} + 20$$

$$\frac{\mathrm{d}f}{\mathrm{d}t} = \frac{f_{inf} - f}{\tau_f}$$

$$f2_{inf} = \frac{0.67}{1 + exp\Big(\dfrac{V + 35}{7}\Big)} + 0.33$$

$$\tau_{f2} = 562 \cdot exp\Big(\frac{-(V + 27)^2}{240}\Big) + \frac{31}{1 + exp\Big(\dfrac{25 - V}{10}\Big)} + \frac{80}{1 + exp\Big(\dfrac{V + 30}{10}\Big)}$$

$$\frac{\mathrm{d}f2}{\mathrm{d}t} = \frac{f2_{inf} - f2}{\tau_{f2}}$$

$$fCaSS_{inf} = \frac{0.6}{1 + \Big(\dfrac{[Ca]_{SS}}{0.05}\Big)^2} + 0.4$$

$$\tau_{fCaSS} = \frac{80}{1 + \left(\frac{[Ca]_{SS}}{0.05}\right)^2} + 2$$

$$\frac{\mathrm{d}fCaSS}{\mathrm{d}t} = \frac{fCaSS_{inf} - fCaSS}{\tau_{fCaSS}}$$

$$I_{CaL} = g_{CaL} \cdot d \cdot f \cdot f2 \cdot fCaSS \cdot 4 \cdot (V - 15) \cdot \frac{F^2}{R \cdot T} \cdot \frac{0.25 \cdot [Ca]_{SS} \cdot exp\Big(2 \cdot (V-15) \cdot \frac{F}{R \cdot T}\Big) - [Ca]_e}{exp\Big(2 \cdot (V-15) \cdot \frac{F}{R \cdot T}\Big) - 1}$$

T-type calcium current

$$d_{Tinf} = \frac{1}{1 + exp\Big(\frac{-23 - V}{6.1}\Big)}$$

$$\tau_{d_T} = 0.6 + \frac{5.4}{1 + exp(0.03 \cdot (V + 100))}$$

$$\frac{\mathrm{d}d_T}{\mathrm{d}t} = \frac{d_{Tinf} - d_T}{\tau_{d_T}}$$

$$f_{Tinf} = \frac{1}{1 + exp\Big(\frac{V + 75}{6.6}\Big)}$$

$$\tau_{f_T} = 1 + \frac{40}{1 + exp(0.08 \cdot (V + 65))}$$

$$\frac{\mathrm{d}f_T}{\mathrm{d}t} = \frac{f_{Tinf} - f_T}{\tau_{f_T}}$$

$$I_{CaT} = g_{CaT} \cdot d_T \cdot f_T \cdot (V - E_{Ca} + 75)$$

Sodium-calcium exchanger current

$$I_{NaCa} = k_{NaCa} \cdot \frac{exp\Big(\gamma \cdot V \cdot \frac{F}{R \cdot T}\Big) \cdot [Na]_i^3 \cdot [Ca]_e - exp\Big((\gamma - 1) \cdot V \cdot \frac{F}{R \cdot T}\Big) \cdot [Na]_e^3 \cdot [Ca]_i \cdot \alpha}{(Km_{Nai}^3 + [Na]_e^3) \cdot (Km_{Ca} + [Ca]_e) \cdot \Big(1 + K_{sat} \cdot exp\Big((\gamma - 1) \cdot V \cdot \frac{F}{R \cdot T}\Big)\Big)}$$

Sodium-potassium pump current

$$I_{NaK} = \frac{p_{NaK} \cdot \frac{[K]_e}{[K]_e + K_{mk}} \cdot \frac{[Na]_i}{[Na]_i + K_{mNa}}}{1 + 0.1245 \cdot exp\Big(-0.1 \cdot V \cdot \frac{F}{R \cdot T}\Big) + 0.0353 \cdot exp\Big(-V \cdot \frac{F}{R \cdot T}\Big)}$$

Transient outward potassium current

$$s_{inf} = \frac{1}{1 + exp\Big(\frac{V + 20}{5}\Big)}$$

$$\tau_s = 85 \cdot exp\Big(\frac{-(V + 45)^2}{320}\Big) + \frac{5}{1 + exp\Big(\frac{V - 20}{5}\Big)} + 3$$

$$\frac{\mathrm{d}s}{\mathrm{d}t} = \frac{s_{inf} - s}{\tau_s}$$

$$r_{inf} = \frac{1}{1 + exp\Big(\frac{20 - V}{6}\Big)}$$

$$\tau_r = 9.5 \cdot exp\Big(\frac{-(V + 40)^2}{1800}\Big) + 0.8$$

$$\frac{\mathrm{d}r}{\mathrm{d}t} = \frac{r_{inf} - r}{\tau_r}$$

$$I_{to} = g_{to} \cdot r \cdot s \cdot (V - E_K)$$

Funny current

$$y_{inf} = \begin{cases} 0.01329 + \dfrac{0.99921}{1 + exp\left(\dfrac{V + 97.134}{8.1752}\right)}, & V < -80\,mV \\ 0.0002501 \cdot exp\left(\dfrac{-V}{12.861}\right), & \text{otherwise} \end{cases}$$

$$\tau_y = \frac{1}{0.00036 \cdot \dfrac{V + 148.8}{exp(0.066 \cdot (V + 148.8)) - 1} + 0.0001 \cdot \dfrac{V + 87.3}{1 - exp(-0.2 \cdot (V + 87.3))}} - 54$$

$$\frac{\mathrm{d}y}{\mathrm{d}t} = \frac{y_{inf} - y}{\tau_y}$$

$$g_{fNa} = 0.5949 \cdot g_{fK}$$

$$I_{fNa} = g_{fNa} \cdot y \cdot (V - E_{Na})$$

$$I_{fK} = g_{fK} \cdot y \cdot (V - E_K)$$

$$I_f = I_{fNa} + I_{fK}$$

Calcium pump current

$$I_{pCa} = g_{pCa} \cdot \frac{[Ca]_i}{[Ca]_i + K_{pCa}}$$

Potassium pump current

$$I_{pK} = g_{pK} \cdot \frac{V - E_K}{1 + exp\left(\dfrac{25 - V}{5.98}\right)}$$

Sodium background current

$$I_{bNa} = g_{bNa} \cdot (V - E_{Na})$$

Calcium background current

$$I_{bCa} = g_{bCa} \cdot (V - E_{Ca})$$

Calcium concentration

$$I_{rel} = a_{RyR} \cdot V_{rel} \cdot O \cdot ([Ca]_{SR} - [Ca]_{SS})$$

$$I_{up} = a_{SERCA} \cdot \frac{Vmax_{up}}{1 + \dfrac{K_{up}^2}{[Ca]_i^2}}$$

$$I_{leak} = a_{RyR} \cdot V_{leak} \cdot ([Ca]_{SR} - [Ca]_i)$$

$$I_{xfer} = V_{xfer} \cdot ([Ca]_{SS} - [Ca]_i)$$

$$O = k1 \cdot [Ca]_{SS}^2 \cdot \frac{R_{prime}}{k3 + k1 \cdot [Ca]_{SS}^2}$$

$$\frac{\mathrm{d}R_{prime}}{\mathrm{d}t} = -k2 \cdot [Ca]_{SS} \cdot R_{prime} + k4 \cdot (1 - R_{prime})$$

$$k1 = \frac{k1_{prime}}{kcasr}$$

$$k2 = k2_{prime} \cdot kcasr$$

$$kcasr = max_{SR} - \frac{max_{SR} - min_{SR}}{1 + \left(\frac{EC}{[Ca]_{SR}}\right)^2}$$

$$Ca_{i_{bufc}} = \frac{1}{1 + a_{TropC} \cdot Buf_c \cdot K_{buf_c}} \cdot ([Ca]_i + K_{buf_c})^2$$

$$Ca_{SR_{bufSR}} = \frac{1}{1 + a_{CASQ} \cdot Buf_{SR} \cdot K_{buf_{SR}}} \cdot ([Ca]_{SR} + K_{buf_{SR}})^2$$

$$Ca_{SS_{bufSS}} = \frac{1}{1 + a_{TropC} \cdot Buf_{SS} \cdot K_{buf_{SS}}} \cdot ([Ca]_{SS} + K_{buf_{SS}})^2$$

$$\frac{\mathrm{d}[Ca]_i}{\mathrm{d}t} = Ca_{i_{bufc}} \cdot \left((I_{leak} - I_{up}) \cdot \frac{V_{SR}}{V_c} + I_{xfer} - \frac{(I_{bCa} + I_{pCa} - 2 \cdot I_{NaC}) \cdot C_m}{2 \cdot V_c \cdot F}\right)$$

$$\frac{\mathrm{d}[Ca]_{SR}}{\mathrm{d}t} = Ca_{SR_{bufSR}} \cdot (I_{up} - (I_{rel} + I_{leak}))$$

$$\frac{\mathrm{d}[Ca]_{SS}}{\mathrm{d}t} = Ca_{SS_{bufSS}} \cdot \left(\frac{-(I_{CaL} + I_{CaT}) \cdot C_m}{2 \cdot V_{SS} \cdot F} + I_{rel} \cdot \frac{V_{SR}}{V_{SS}} - I_{xfer} \cdot \frac{V_c}{V_{SS}}\right)$$

Sodium concentration

$$\frac{\mathrm{d}[Na]_i}{\mathrm{d}t} = \frac{-(I_{Na} + I_{fNa} + I_{bNa} + 3 \cdot I_{NaK} + 3 \cdot I_{NaCa})}{V_c \cdot F} \cdot C_m$$

Potassium concentration

$$\frac{\mathrm{d}[K]_i}{\mathrm{d}t} = \frac{-(I_{K1} + I_{to} + I_{Kr} + I_{Ks} + I_{fK} + I_{pK} + I_{stim} - 2 \cdot I_{NaK})}{V_c \cdot F} \cdot C_m$$

Model parameters

Physical constants

R	8.314	$J/(mol \cdot K)$
T	310	K
F	96.485	$C/mmol$

ODEs initial conditions

V	-82.31905038433626	mV
m	0.003168041765753429	
h	0.6632395003358482	
j	0.5311135520174594	
$Xr1$	0.07425181787778348	
$Xr2$	0.4412585279437558	
Xs	0.021216229948985553	
d	$4.966231702796764 \times 10^{-5}$	
f	0.6740117689220396	
$f2$	0.9095881370387117	
$fCaSS$	0.9867658763427091	
d_T	$5.93224113564556 \times 10^{-5}$	
f_T	0.7567010784040331	
s	0.999996217592982	
r	$3.8729744057242666 \times 10^{-8}$	
y	0.02706175213059363	
$[Ca]_i$	0.00011161967314543063	mM
$[Ca]_{SS}$	0.0005689010815000564	mM
$[Ca]_{SR}$	11.071118801447446	mM
R_{prime}	0.9265739700622043	
$[Na]_i$	4.765748992328347	mM
$[K]_i$	140.46186062237774	mM

Cell geometry

C_m	11.823	pF
V_c	348.677	μm^3
V_{SS}	0.312	μm^3
V_{SR}	6.164	μm^3

Stimulus current

start	10	ms
period	353	ms
duartion	1	ms
amplitude	52	pA/pF

Ion external concentrations

$[Na]_e$	140	mM
$[K]_e$	5.4	mM
$[Ca]_e$	2	mM

Ion channels and transporters conductance

g_{Na}	7.627	nS/pF
g_{K1}	2.785	nS/pF
g_{Kr}	0.152	nS/pF
g_{Ks}	0.375	nS/pF
g_{CaL}	1.087×10^{-2}	$L/(F \cdot s)$
g_{CaT}	1.587	nS/pF
k_{NaCa}	681.377	pA/PF
p_{NaK}	3.998	pA/pF
g_{to}	5.719×10^{-2}	nS/pF
g_{fK}	0.113	nS/pF
g_{pCa}	0.136	nS/pF
g_{pK}	0.595×10^{-2}	nS/pF
g_{bNa}	1.598×10^{-4}	nS/pF
g_{bCa}	3.959×10^{-4}	nS/pF

Calcium dynamics

$k1_{prime}$	0.15	$mM^{-2} \cdot ms^{-1}$
$k2_{prime}$	0.045	$(mM \cdot ms)^{-1}$
$k3$	0.06	ms^{-1}
$k4$	0.005	ms^{-1}
EC	1.5	mM
max_{SR}	2.5	
min_{SR}	1	
V_{rel}	0.102	ms^{-1}
V_{xfer}	0.0038	ms^{-1}
K_{up}	0.00025	mM
V_{leak}	0.00036	ms^{-1}
$Vmax_{up}$	0.006375	$(mM \cdot ms)^{-1}$
Buf_c	0.2	mM
K_{buf_c}	0.001	mM
Buf_{SR}	10	mM
$K_{buf_{SR}}$	0.3	mM
Buf_{SS}	0.4	mM
$K_{buf_{SS}}$	0.00025	mM
a_{TropC}	0.868	
a_{CASQ}	0.542	
a_{SERCA}	0.994	
a_{RyR}	0.239	

Other parameters

P_{KNa}	0.03	
K_{mk}	1	mM
K_{mNa}	40	mM
K_{sat}	0.1	
α	2.5	
γ	0.35	
Km_{Ca}	1.38	mM
Km_{Nai}	87.5	mM
