# Supplementary material for: A model for the human fetal ventricular myocyte electrophysiology
Source: PLoS Comput Biol. 2026 Jan 27;22(1):e1013889. doi: 10.1371/journal.pcbi.1013889 (PMC12863696; doi:10.1371/journal.pcbi.1013889)
Supplement: S2 Text — (PDF) [file pcbi.1013889.s002.pdf]

Human fetal T-type calcium current model

1 Introduction

T-type channels (from "transient" and "tiny") are a specific type of calcium channels which are activated at rather negative membrane potentials, within the range of -50 to $-60\,mV$, and are rapidly inactivated, in contrast to high threshold and slow activation of L-type channels which are normally activated by cell depolarizations of $-30\,mV$ [1, 2, 3, 4]. Another characteristic of the T-type calcium current is that in the I-V diagram it reaches its peak at negative voltages in contrast with L-type current that typically showed a peak current at zero or positive voltages. Three varieties of T-type channel have been identified so far, Cav3.1, Cav3.2 and Cav3.3 respectively (or α1G, α1H and α1I) [5, 6]. Cardiac T-type current recordings in literature were performed by Bkaily (1992 embryonic chick and human fetal ventricular myocites) [7], Nilius (1986, guinea pig sino-atrial node) [1], Hagiwara et al. (1988, rabbit sino-atrial node), Kawano and DeHaan (1989, embryonic chick apical ventricular myocytes) [8], Fermini and Nathan (1991, rabbit sino-atrial node) [9], Furukawa et al. (1992, neonatal rat ventricular myocytes), Maylie and Morad (1995, shark ventricular myocytes) [10], Cribbs et al. (1998, human embryonic kidney (293) cells transfected with α1H channels cloned from human heart) [4], Zhou and January (1998, canine Purkinje cells) [11], Yeoman et al. (1999, Lymnaea ventricular myocytes) [12]. In all cases the T-type current were obtained as the difference between the total calcium current, elicited from a holding potential of $-80/-90\,mV$, and the L-type calcium current, obtained from a holding potential of $-40/-50\,mV$. For a comprehensive review on T-type channels refer to [6].
Experimental results suggest that T-type calcium current is important in supporting ventricular contraction in early development when the function of the sarcoplasmatic reticulum is not well developed and when L-type current is low [13, 14, 15]. Furthermore, the expression of T-type channels is significantly higher in immature than mature cardiomyocyte and disappears in adult, unlike L-type channels whose expression increases during heart development until adult age [15, 16, 14].

**Heart**	**Numeration in [17]**	**Week of gestation [w+d]**	**Abnormality**	**Number of cells**	**Notes**
heart 1	2	13	trisomy 13	5	
heart 2	3	12	trisomy 18	3	
heart 3	4	16+6	trisomy 21	2	*

Table A: Characteristics of the collected fetal hearts.* isolated as atrial and ventricular cells.

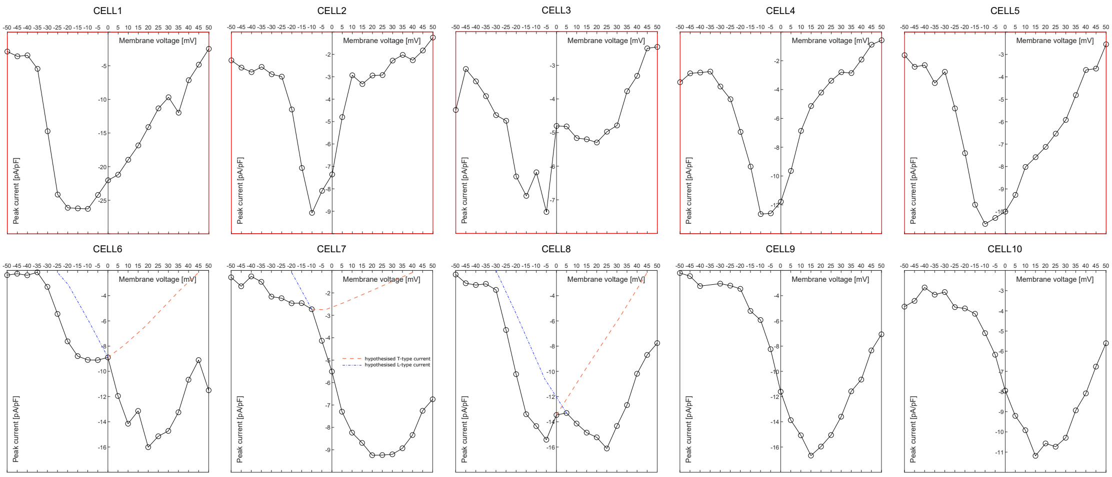

Figure A: Peak calcium current on voltage of experimental recordings. The upper row shows the cells selected for the model while the lower row shows the discarded cells. The red and blue lines are meant to suggest the possible behaviour of T-type and L-type currents respectively to highlight the L-type current artifact.

2 Methods

2.1 Experimental measurements

Human fetal cardiomyocytes were isolated from tissues obtained from fetuses aged 12–17 weeks terminated (STOP, surgical termination of pregnancy) due to genetic abnormalities (Trisomy 13, 18 and 21) [17, 18]. A total of 7 hearts were obtained from which cells were isolated successfully. Three of these hearts were used to perform T-type calcium current recording (Table A in S2 Text).

The cells were incubated in a $6\,mM$ Ca^{2+}-TEA-Cl^{-}based solution containing nifedipine (see Table B in S2 Text for concentration) to suppress the L-type calcium current with minimal effects on T-type current. A calcium chelator (EGTA, $5\,mM$) was used to bind excess intracellular calcium. Although it was not possible to measure internal calcium concentration directly, Ca^{2+}concentration inside the cell is assumed to be held at representative diastolic concentrations, in the region of $100\,nM$. T-type calcium currents from 10 different cells coming from 3 different hearts were recorded (Figure A in S2 Text) at room temperature (20-22 degrees) and the cell membrane capacitance was measured (see Table B in S2 Text). Currents were elicited in voltage-clamp protocol from a holding potential of $-90\,mV$ to test potentials ranging from -50 to $+50\,mV$ in $5\,mV$ increments over $150\,ms$. In some cases the nifedipine did not succeed in fully blocking the L-type current. Three cells of heart 1 showed a significant non-zero L-type current as well as one of the cells of heart 2. In another cell of heart 2 the recording of L-type current was not performed but the peak currents curve on voltage presented the same behavior of the cells with non-zero L-type current, i.e. the maximum calcium current was detected at positive voltages around $15\,mV$ (see Figure A in S2 Text). This is characteristic of L-type calcium current traces and inconsistent with T-type current recordings in literature which all, as well as the other cells of our experiment, show a peak current at negative voltages less than $-10\,mV$ (see section 1). For this reason the cells mentioned above were excluded from the model fitting. The cells selection process is shown in Figure B in S2 Text.

2.2 Mathematical model

2.2.1 T-type calcium current

Mathematical models of T-type calcium current present in literature are mostly based on the experimental data of Nilius [1], Hagiwara et al. [2] and Fermini and Nathan [9]. Well-known models for the T-type current are Wilders et al. (1991, rabbit sinoatrial node) [19], based on data of Hagiwara, Demir et al. (1994, rabbit sino-atrial node) [20], based on data of Hagiwara, Nilius

Heart	Cell	Membrane capacitance [pF]	Nifedipine [μM]	Maximum current voltage [mV]	T-type current (max) [pA]	L-type current (max) [pA]
heart 1	cell 1	13.5	100	-15	350	~ 0
	cell 2	12		+20	200	120
	cell 3	15		+30	140	100
	cell 4	10.5	100	+25	170	80
	cell 5	16		-10	150	-
heart 2	cell 6	9	100	+15	150	-
	cell 7	10		+20	100	70
	cell 8	10.2		-15	90	-
heart 3	cell 9	20	200	-10	250	~ 0
	cell 10	16		-10	200	-

Table B: Characteristics of the cells analyzed in the experiment. - : not measured

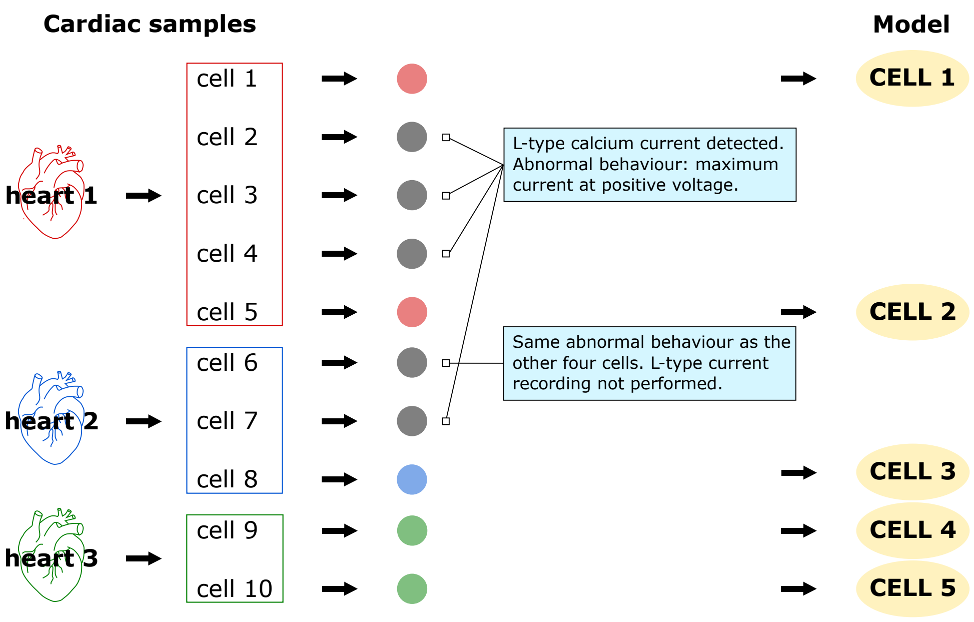

Figure B: Criteria of cells selection for the model.

and Fermini and Nathan, Dokos et al. (1996, sinoatrial node) [21], adapted from Wilders, and Korhonen et al. (2009, rat neonatal ventricular myocytes) [22], adapted from Dokos et al. . There is not a fetal or adult human T-type calcium channel model that provides a single starting point for creating a model. Here we aim to build a model that is as simple as possible. We consider the two model structures initially proposed by Demir and Dokos. Demir et alȧdopted a classic Hodgkin-Huxley formalism to model T-type current and its equations for the gating variables are based on data from Nilius (25°C) as well as Hagiwara and Fermini and Nathan (37°C) and the work of adjustment of the experimental parameters to be suitable for the same temperature of 37°C was done. Dokos et al. mantained the same equations as Wilders for the gating variables, fitted to data of Hagiwara consistently recorded at 37°C, but used a Hodjkin-Huxley formulation for the T-type current instead of the Goldmann-Hodgkin-Katz utilised by Wilders, so it was preferable for reasons of simplicity and coherence with Demir.

2.2.2 L-type calcium current

For some of the cells analysed, especially CELL 3 and 5, the T-type current model alone was not sufficient to obtain a good fitting with the experimental current traces which exhibit some of the characteristics of L-type current. For this reason an additional fitting was performed using a model which accounts of both T-type and L-type currents. Formulations for the L-type current of either Dokos and Demir models were considered as well as the L-type calcium channel model of Ten Tusscher 2004 for human ventricular myocyte[23], which was used as a reference human L-type calcium model.

2.2.3 Units and parameters

Time is expressed in ms, voltage in mV, ionic concentration in mM, ionic current in pA/pF, and channel conductance in nS/pF as intended divided by the cell capacitance which is in pF. All the equations parameters are taken the same as in the original models except for the channel conductance which is taken as the average of fitted conductance of the five cells. Cell capacitance in the model is taken the average of cell capacitance of all the ten cells of the experiment (see Table B in S2 Text). Internal as external calcium concentration are as in section 2.1.

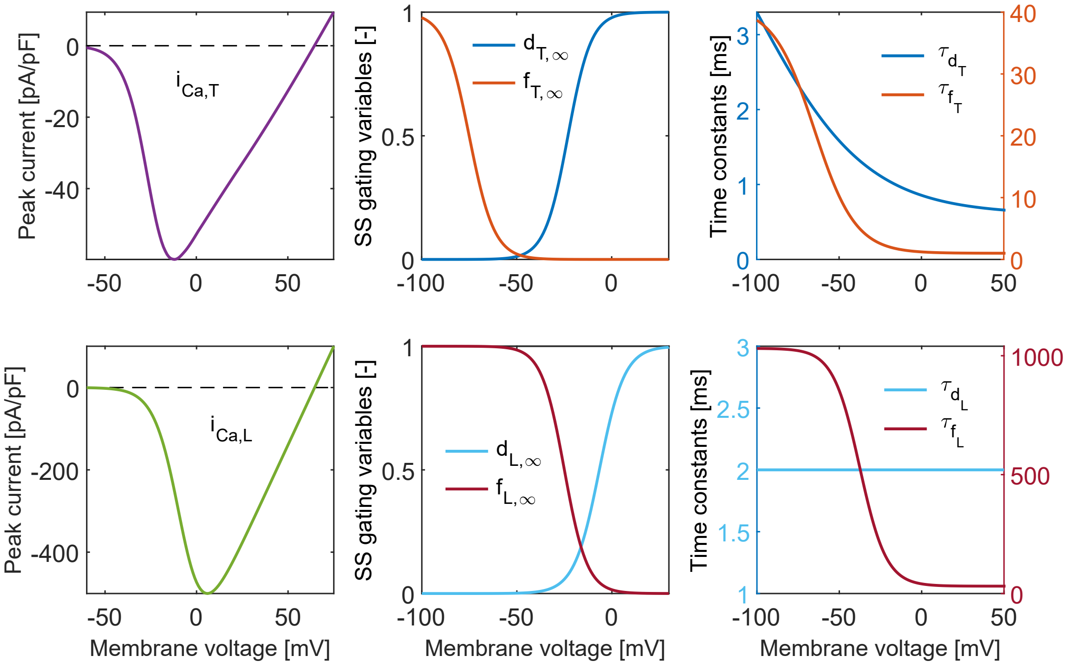

Figure C: I-V curve and gating variables as in Dokos et al. [21] . Both T-type (top) and L-type (bottom) currents dynamics are illustrated. Conductance values for T-type and L-type currents used for the simulation are as in Dokos.

2.2.4 Model equations

Dokos model In Dokos et al. the T-type calcium current is modelled following a standard Hodgkin-Huxley's current formulation:

$$i_{Ca,T} = G_{Ca,T}\, d_T\, f_T\, (V - E_{rev,T})$$

where $G_{Ca,T}$ is the channel maximum conductance and gating variables d_T, f_T of activation and inactivation, respectively, varies on time according to:

$$\frac{\mathrm{d}d_T}{\mathrm{d}t} = \frac{d_{T,\infty} - d_T}{\tau_{d_T}}, \qquad \frac{\mathrm{d}f_T}{\mathrm{d}t} = \frac{f_{T,\infty} - f_T}{\tau_{f_T}},$$

where $d_{T,\infty}$, $f_{T,\infty}$ are the steady-state gating variables, depending only on voltage, as well as the gating time constants (tau) τ_{d_T} and τ_{f_T} (Figure C in S2 Text).
Equations for L-type calcium are described by:

$$i_{Ca,L} = G_{Ca,L}\, d_L\, f_L\, f_{2L}\, (V - E_{rev,L})\,,$$

with a second inactivation gate f_{2L} depending only on the internal concentration $[Ca]_i$.
For both T-type and L-type currents, the reversal potential is taken as the sum of Nernst potential for calcium E_{Ca} and a constant voltage. This constant was in a first step introduced in L-type current model in order to compensate the discrepancy between the Nernst potential for calcium and the reversal potential in experimental measurements of L-type current carried out in several studies. This discrepancy is due to a possible permeability of L-type channel to $Na^+$and K^+or a contamination of the measurements by Na^+-Ca^{2+}exchange current (for a better understanding of this point see [21]). The same constant was utilised then to model T-type calcium current as well because experimental evidence shows that both currents, L-type and T-type, have about the same dependence on the extracellular calcium concentration [2, 19].

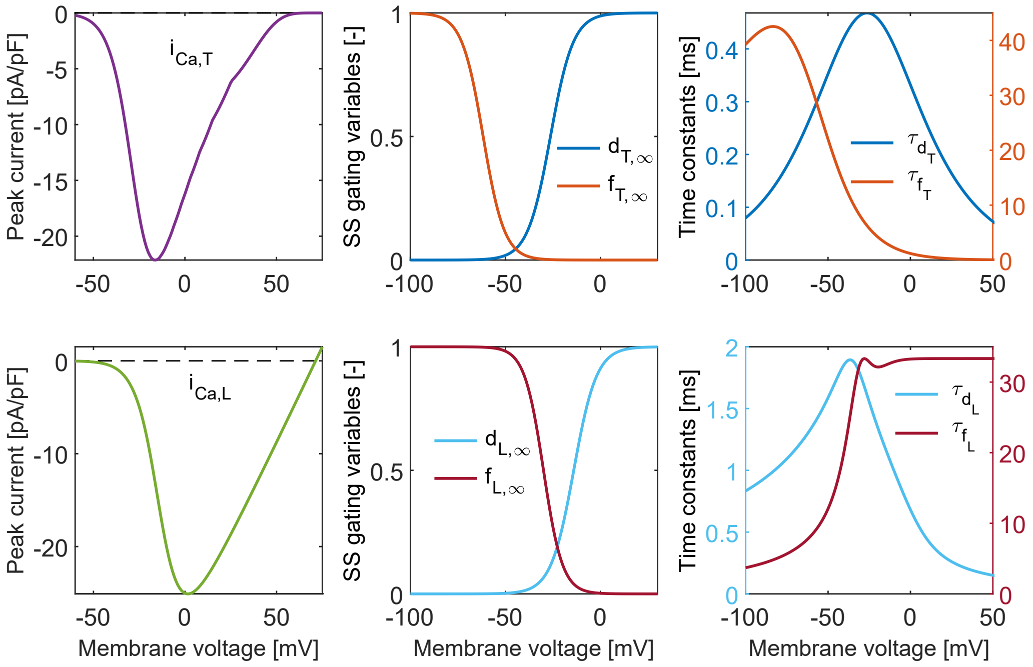

Figure D: I-V curve and gating variables as in Demir et al. [20]. T-type (top) and L-type (bottom) currents. Conductance values for T-type and L-type currents used for the simulation are as in Demir.

Demir model T-type calcium current is modelled as in Dokos et al.:

$$i_{Ca,T} = G_{Ca,T}\, d_T\, f_T\, (V - E_{rev,T})\,,$$

while L-type formulation is adjusted by an addition coefficient:

$$i_{Ca,L} = G_{Ca,L} \left(d_L \, f_L + 0.095 d_{L,\infty} \right) \left(V - E_{rev,L} \right).$$

In Demir model (Figure D in S2 Text) the problem of compensating for the difference between the reversal potential and the Nernst potential (see discussion above) had been solved considering a constant reversal potential, independent from calcium concentration and evaluated by experimental measurements.

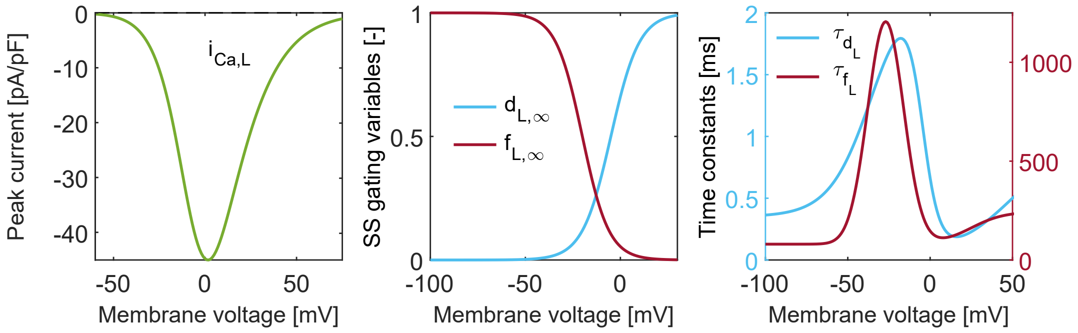

Figure E: I-V curve and gating variables of L-type current modelled as in Ten Tusscher et al. [23]. Conductance used for the simulation as in Ten Tusscher.

Ten Tusscher model In Ten Tusscher only L-type calcium channel is present and he driving force of the current is modeled with a Goldmann-Hodgkin-Katz equation as follows:

$$i_{Ca,L} = G_{Ca,L} \, d_L \, f_L \, f_{2L} 4 \frac{VF^2}{RT} \frac{[Ca]_i e^{\frac{2VF}{RT}} - 0.341[Ca]_e}{e^{\frac{2VF}{RT}} - 1},$$

where d_L and f_L are the voltage-dependent activation and inactivation gate respectively (Figure E in S2 Text) and f_{2L} is an intracellular calcium-dependent inactivation gate.

Voltage clamp protocols for calcium currents simulated with the three model at comparison is shown in Figure F in S2 Text.

2.3 Fitting of the model to experimental data

The current traces were imported in Matlab as current vectors and time vector. Five cells in total were analyzed: CELL 1,2 from heart 1, and CELL 3 from heart 2 and CELL 4,5 from heart 3 (see Figure B in S2 Text). The recorded current from each cell was divided by its respective cell capacitance (Table B in S2 Text). To filter out the noise from the curves, the noise amplitude was computed in the current curve tail and subtracted by the upper peaks and added to the lower peaks. This procedure was applied untill the noise was reduced of 75%.
In whole-cell membrane current recording, the current response is affected by the recording system. When a step voltage change is applied, the transfer of the signal path is delayed by a few hundred microseconds. This requires proper adjustment of the zero time of the recorded current relative to the zero time of the step voltage onset for up to the introduced delay [24]. While it is possible to calibrate for this [24], the experimental parameters were not recorded during measurements. Therefore, we introduce the onset time as a free variable in the fitting. Any curve (any cell at any voltage step) was fitted with any of the eight model combinations considered: T-Demir, T-Demir L-Demir, T-Demir L-Dokos, T-Demir L-TenTusscher, T-Dokos, T-Dokos L-Demir, T-Dokos L-Dokos, T-Dokos L-TenTusscher. The fit consisted in finding the conductance which minimize the distance between experimental and simulated current curves for each individual or pair of channel types. The conductance was made varying in a confidence interval and for each of its values the current traces with imposed conductance were fitted with the current equationusing as fitting parameter the onset time t_0. The fit was performed in matlab using *lsqcurvefit* matlab function (method: non linear least square, algorithm: trust-region-reflective, initial guess: $t_0 = 0$). For any single voltage the error between experimental

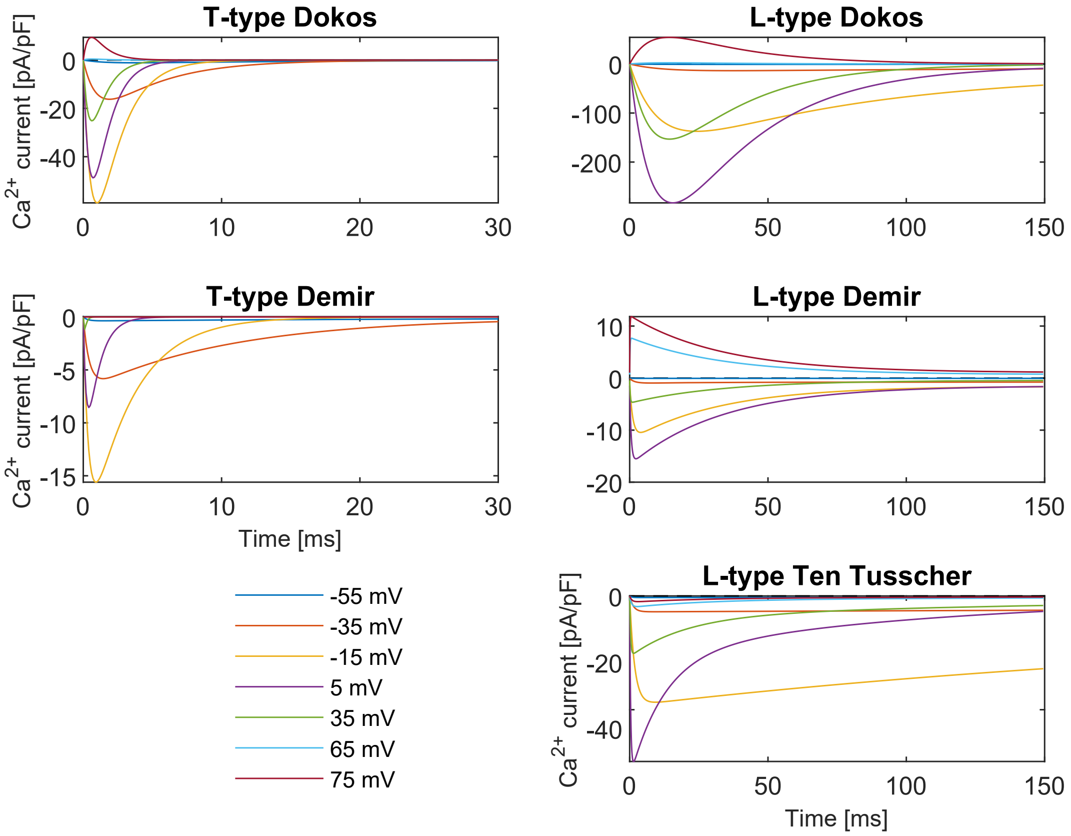

[h!]

Figure F: Comparison of calcium current on time simulated at different voltages for the three models considered. T-type (left column) and L-type (right column). Conductances used for the simulation are as in the respective models.

and simulated curve was computed has the L^2 norm of the difference of the curves, normalized the L^2 norm of the experimental curve:

$$E(V_j) = \frac{\sqrt{\sum_i (I_{exp}(t_i) - I_{sim}(t_i))^2}}{\sqrt{\sum_i (I_{exp}(t_i))^2}}$$

The total error for a single cell was then computed as the weighted average on voltage steps of the single voltage step error, where the weight was chosen as the normalized peak current, in order to prioritise fitting to traces with a better signal to noise ratio, as follows:

$$E_{tot} = \sum_j \frac{1}{v} w_j E(V_j) ,$$

$$w_j = \frac{I_{peak}(V_j)}{\max_j I_{peak}(V_j)} ,$$

where v is the number of voltage steps (21). After computing the total error for any conductance in the interval, the one (or the couple T-L in case of mixed model) which produced the minimum error was chosen as the final conductance for the correspondent cell and model.

3 Results and discussion

Calcium current of five cells was analysed and fitted with eight different combinations of T-type and L-type models from Demir, Dokos and Ten Tusscher. The errors for each cell, for each model permutation, are presented in Figure J in S2 Text. Figure G in S2 Text shows the best fitted T-type model for each cell. Current traces fit results and the computed error indicates that the best fit of T-type calcium current is obtained using Dokos T-type model for CELL 1 and Demir model for CELL 2 to 5. The fit obtained is good in both cases. The peak current curve (B) for CELL 1 shows a very good agreement between the model and experimental data, as for the reversal potential value as for the peak voltage. CELL 2 to 4 simulated peak current curve shows a good correspondence with experimental data for the reversal potential. The simulated peak voltage is more negative than the real one but this is due in part to the fact that the only parameter we are fitting is the channel conductance while the steady state state gating variable are taken from the original model and are fixed, in part to the error left by the removal of L-type current contribution which CELL 2 to 5 are more affected by than CELL 1.

3.1 T-type calcium current

Figures H and I in S2 Text shows the models comparison results obtained respectively for CELL 1 and CELL 3, as representative of the second group of cells. CELL 1 shows different behaviour than other cells. Specifically, the calcium current reaches its reversal potential at a voltage higher than $50\,mV$ while other cells currents reach it at voltages close to $40\,mV$. Current traces of CELL 1 are characterised by a smaller time of decay at negative voltages than current of CELL 2 to 5 and a higher one at positive voltages. CELL 1 presents also a higher current intensity, maximum current of $25\,pA/pF$, compared to other cells where the maximum current is near $10\,mV$. These characteristics resemble those of T-type Dokos model (see Figures C, F and G in S2 Text). CELL 2 to 5 behaviour is on the contrary more in according to Demir T-type model (see Figures D, F and G in S2 Text). For this reason it was decided to build two models for the human fetal T-type calcium current: one with equations of Dokos et al. and fitted conductance of CELL 1 and the other using Demir T-type model equations and conductance the average of fitted conductances of CELL 2 to 5. This choice is also supported by error computation (see Figure J in S2 Text). Figure K in S2 Text shows the original calcium currents filtered by removing the L-type contribution as obtained with the best fit and best models combination for any single cell, then the resulting currents where averaged among the 5 cells for any voltage step. This figure is meant to provide an idea of how the T-type caclium current looks like for a human fetal ventricular myocyte aged 12 to 17 gw.

T-type calcium current final model

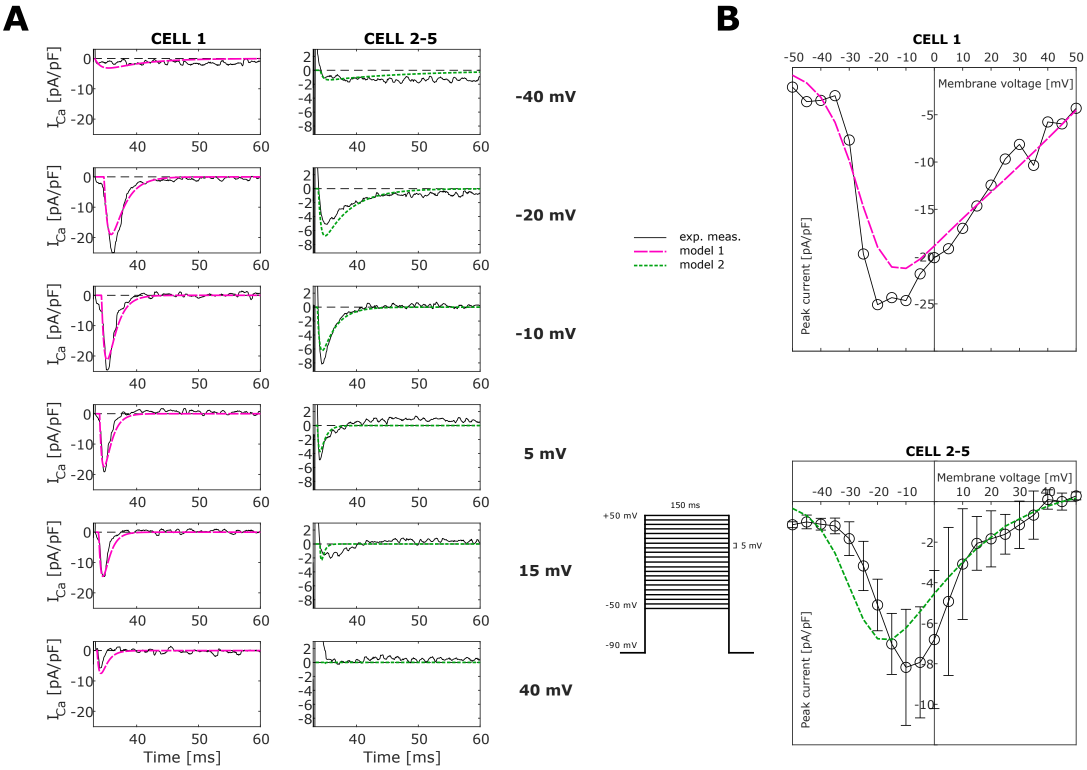

Figure G: A: in the left column the experimental traces from CELL 1 cleared of L-type current are compared with Dokos T-type model with imposed conductance (HFVM model 1) while in the right column the average of cleared experimental traces from CELL 2 to 5 are compared with Demir T-type model with imposed conductance (HFVM model 2). B: same comparison as in A between peak current curves on voltage. The average peak current curve is plotted with its standard deviation bars.

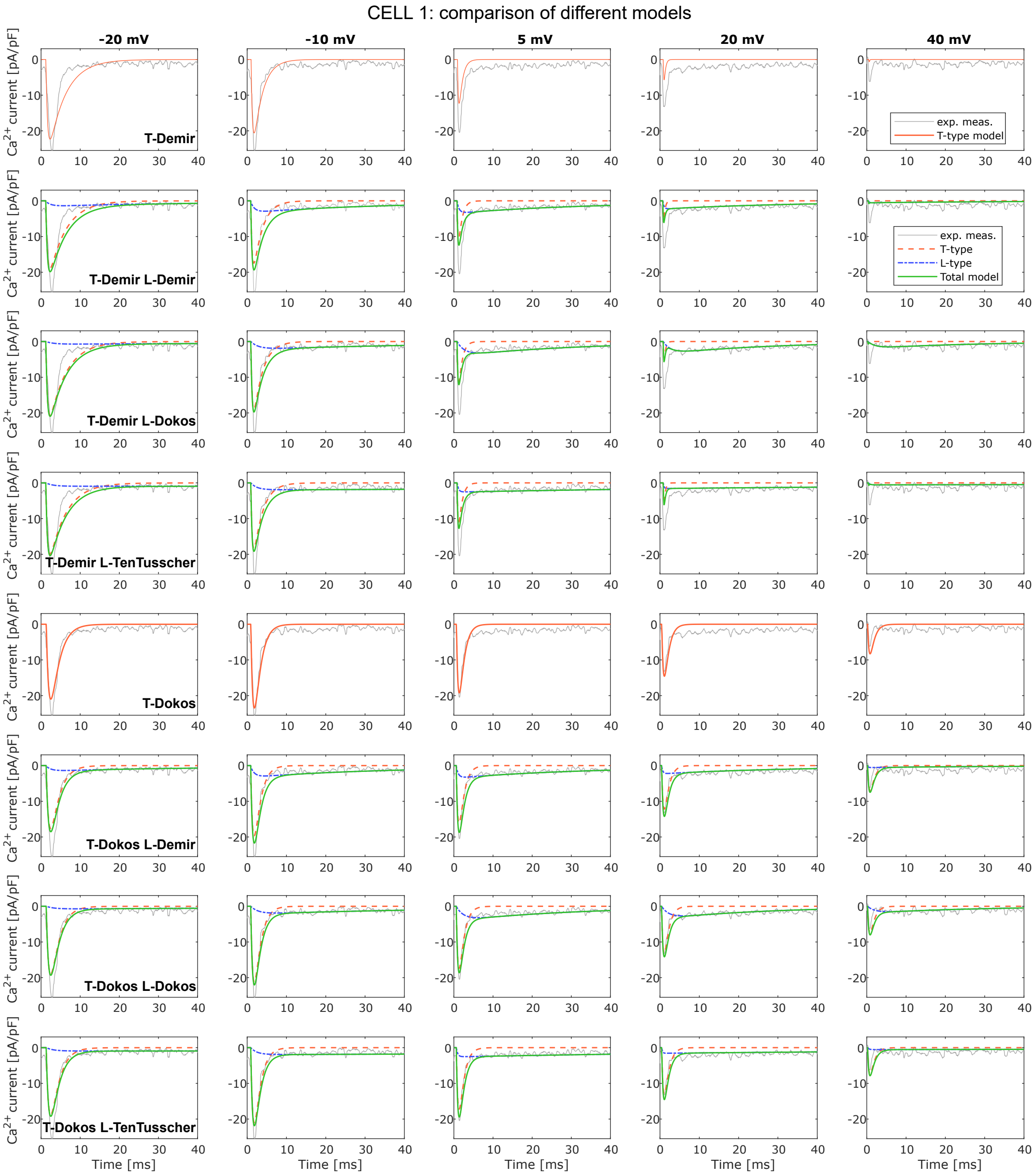

Figure H: CELL 1 calcium current traces fitting: comparison of different models for both T- and L-type calcium current

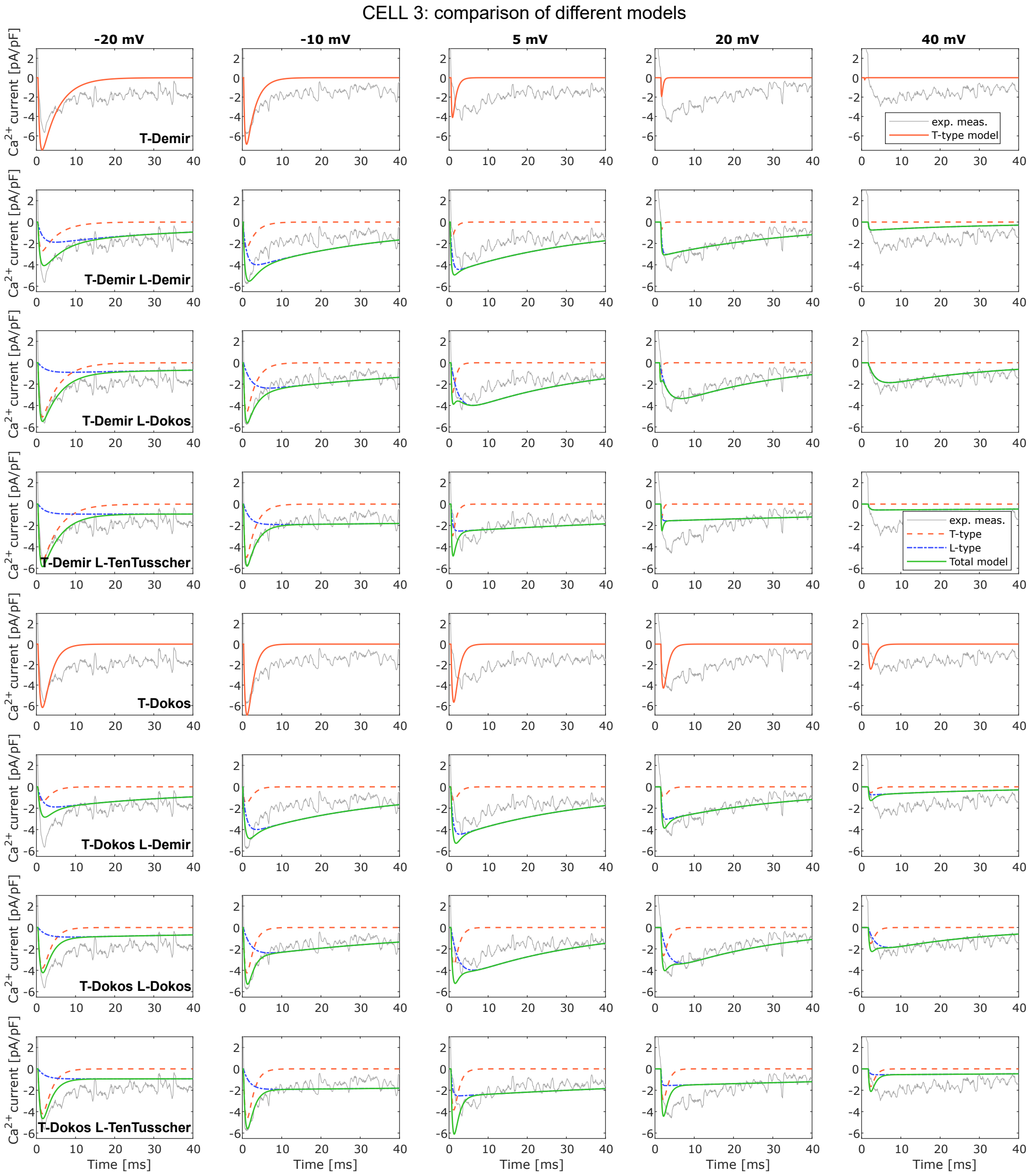

Figure I: CELL 1 calcium current traces fitting: comparison of different models for both T- and L-type calcium current

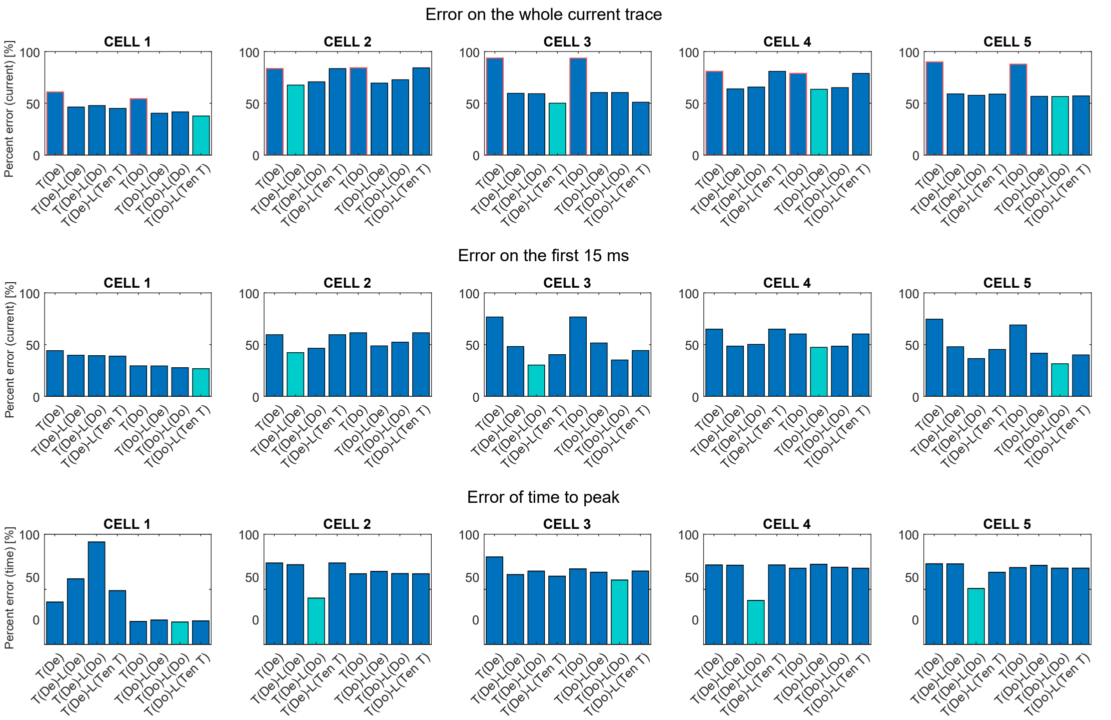

Figure J: Single cells percent error between simulated and experimental calcium current and between respective times to peak: comparison of different models combinations. The red boundaries in the first row indicate the single T-type channel models.

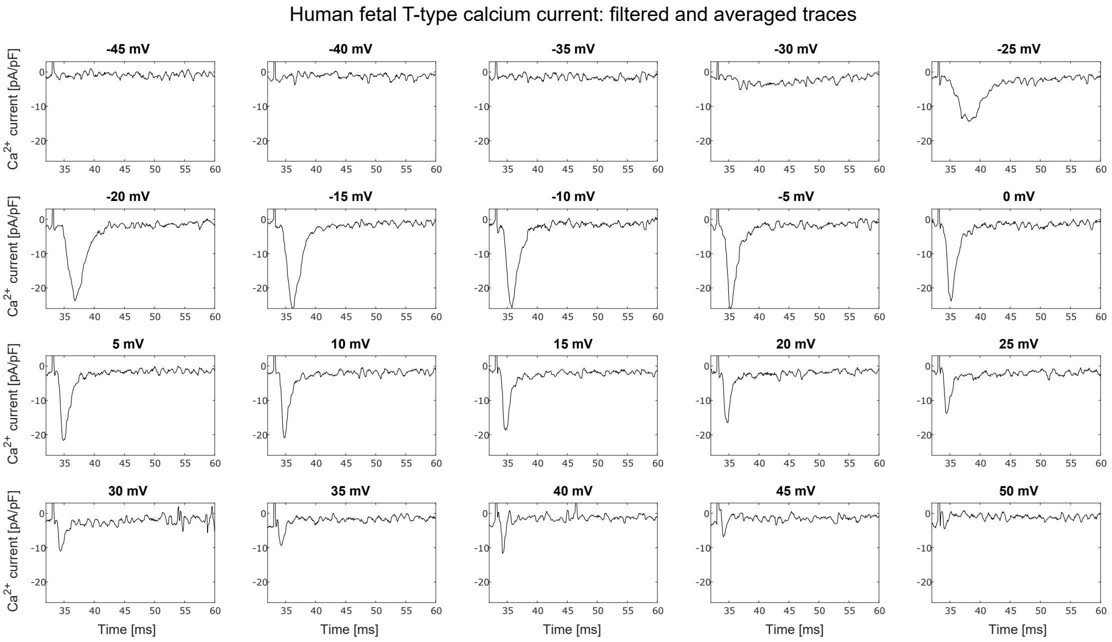

Figure K: The original calcium current where filtered by removing the L-type contribution as obtained with the best fit and best models combination for any single cell, then the resulting currents where averaged among the 5 cells for any voltage step. This figure is meant to provide an idea of how the T-type caclium current looks like for a human fetal ventricular myocyte aged 12 to 17 gw.

3.2 L-type calcium current

It's possible to observe in Figures H and I in S2 Text that the addition of L-type current contribution provided a much better agreement with experimental recordings than considering T-type simulated current alone, especially in case of CELL 2 to 5 whose T-type calcium response is more affected by L-type current interference. This is also confirmed by the computation of the error in Figure J in S2 Text. As for the choice of the L-type model we wanted to have one unique choice for all the cells because we don't have enough information about L-type current in human fetal myocyte since all we have is an interference due to the lack of success in fully blocking L-type channels during the experiment. The Ten Tusscher L-type model exhibits a current almost constant on time for positive voltages due to the long inactivation time (see Figures E and F in S2 Text), with negative steady-state current values. Such characteristic is not seen in fetal cells whose calcium current steady state value is approximately 0. Fitting of CELL 3 and 5, which are the most representative of L-type behaviour since are the most affected, with Ten Tusscher L-type model gives the same results as using T-type models alone. For this reason Ten Tusscher model for L-type current was excluded. As for the choice between Demir and Dokos L-type models it can be observed from Figures H and I in S2 Text that the Dokos model follows more accurately the course of current on time while L-type current traces simulated with Demir model have a too short time of activation compared with the experimental traces and are almost straight lines (see also Figure F in S2 Text). The choice of Dokos for modelling L-type current is also supported by the computation of the time to peak error as it can be seen in Figure J in S2 Text.

4 Conclusion

Experimental recordings of T-type calcium current from five cells coming from three fetal hearts of 12-17 weeks of gestation have been used to build a computational electrophysiological model for the human fetal ventricular myocyte (HFVM) T-type calcium current. Due to the variability of the cells two different models have been obtained. Model 1, which uses the T-type current equations from Dokos et al. and T-type conductance from the fitting of CELL 1 using the combination T-type + L- type Dokos models, and Model 2, with Demir et al. equations and T-type conductance as the average of T-type conductances obtained from currents of CELL 2 to 5 fitted with the combination T-type Demir + L-type Dokos models.

References

[1] B Nilius. “Possible functional significance of a novel type of cardiac Ca channel.” In: *Biomedica biochimica acta* 45.8 (1986), K37–K45.

[2] N Hagiwara, H Irisawa, and M Kameyama. “Contribution of two types of calcium currents to the pacemaker potentials of rabbit sino-atrial node cells.” In: *The Journal of physiology* 395.1 (1988), pp. 233–253.

[3] T Furukawa et al. “Endothelin-1 enhances calcium entry through T-type calcium channels in cultured neonatal rat ventricular myocytes.” In: *Circulation Research* 71.5 (1992), pp. 1242–1253.

[4] LL Cribbs et al. “Cloning and characterization of α1H from human heart, a member of the T-type Ca^{2+}channel gene family”. In: *Circulation research* 83.1 (1998), pp. 103–109.

[5] B Nilius and E Carbone. “Amazing T-type calcium channels: updating functional properties in health and disease”. In: *Pflügers Archiv-European Journal of Physiology* 466.4 (2014), pp. 623–626.

[6] E Perez-Reyes. “Molecular physiology of low-voltage-activated t-type calcium channels”. In: *Physiological reviews* 83.1 (2003), pp. 117–161.

[7] GHASSAN Bkaily et al. “Apamin, a highly potent fetal L-type Ca2+ current blocker in single heart cells”. In: *American Journal of Physiology-Heart and Circulatory Physiology* 262.2 (1992), H463–H471.

[8] S Kawano and RL DeHaan. “Low-threshold current is major calcium current in chick ventricle cells”. In: *American Journal of Physiology-Heart and Circulatory Physiology* 256.5 (1989), H1505–H1508.

[9] B Fermini and RD Nathan. “Removal of sialic acid alters both T-and L-type calcium currents in cardiac myocytes”. In: *American Journal of Physiology-Heart and Circulatory Physiology* 260.3 (1991), H735–H743.

[10] J Maylie and M Morad. “Evaluation of T-and L-type Ca^{2+}currents in shark ventricular myocytes”. In: *American Journal of Physiology-Heart and Circulatory Physiology* 269.5 (1995), H1695–H1703.

[11] Z Zhou and CT January. “Both T-and L-type Ca^{2+}channels can contribute to excitation-contraction coupling in cardiac Purkinje cells”. In: *Biophysical journal* 74.4 (1998), pp. 1830–1839.

[12] MS Yeoman, BL Brezden, and PR Benjamin. “LVA and HVA Ca^{2+}currents in ventricular muscle cells of the *Lymnaea* heart”. In: *Journal of neurophysiology* 82.5 (1999), pp. 2428–2440.

[13] M Artman and WA Coetzee. “Developmental Regulation of Cardiac Ion Channels”. In: *Cardiac Electrophysiology. From Cell to Bedside (Fifth Edition).* Ed. by DP Zipes and J Jalife. Philadelphia: Saunders, 2009. Chap. 15, pp. 157–168.

[14] H Yokoshiki and N Tohse. “Developmental changes in ion channels”. In: *Heart physiology and pathophysiology (Fourth edition).* Ed. by N Sperelakis. Elsevier, 2000. Chap. 41, pp. 719–735.

[15] Y Qu and M Boutjdir. “Gene expression of SERCA2a and L-and T-type Ca channels during human heart development”. In: *Pediatric research* 50.5 (2001), p. 569.

[16] J Liu, Z Laksman, and PH Backx. “The electrophysiological development of cardiomyocytes”. In: *Advanced drug delivery reviews* 96 (2016), pp. 253–273.

[17] F Schultz. “Bile acid signalling in the fetal heart and myometrium”. In: (2015).

[18] O Adeyemi et al. “Ursodeoxycholic acid prevents ventricular conduction slowing and arrhythmia by restoring T-type calcium current in fetuses during cholestasis”. In: *PloS one* 12.9 (2017), e0183167.

[19] R Wilders, HJ Jongsma, and AC Van Ginneken. “Pacemaker activity of the rabbit sinoatrial node. A comparison of mathematical models”. In: *Biophysical journal* 60.5 (1991), pp. 1202–1216.

[20] SS Demir et al. “A mathematical model of a rabbit sinoatrial node cell”. In: *American Journal of Physiology-Cell Physiology* 266.3 (1994), pp. C832–C852.

[21] S Dokos, B Celler, and N Lovell. “Ion currents underlying sinoatrial node pacemaker activity: a new single cell mathematical model”. In: *Journal of theoretical biology* 181.3 (1996), pp. 245–272.

[22] T Korhonen, SL Hänninen, and P Tavi. “Model of excitation-contraction coupling of rat neonatal ventricular myocytes”. In: *Biophysical journal* 96.3 (2009), pp. 1189–1209.

[23] KHWJ Ten Tusscher et al. “A model for human ventricular tissue”. In: *American Journal of Physiology-Heart and Circulatory Physiology* 286.4 (2004), H1573–H1589.

[24] Matej Hot’ka and Ivan Zahradník. “Reconstruction of membrane current by deconvolution and its application to membrane capacitance measurements in cardiac myocytes”. In: *PloS one* 12.11 (2017), e0188452.
